# Supplementary material for: MEK inhibitor resistance in lung adenocarcinoma is associated with addiction to sustained ERK suppression
Source: NPJ Precis Oncol. 2022 Nov 23;6:88. doi: 10.1038/s41698-022-00328-x (PMC9684561; doi:10.1038/s41698-022-00328-x)
Supplement: Supplementary file 1 — Supplementary Figures [file 41698_2022_328_MOESM1_ESM.pdf]

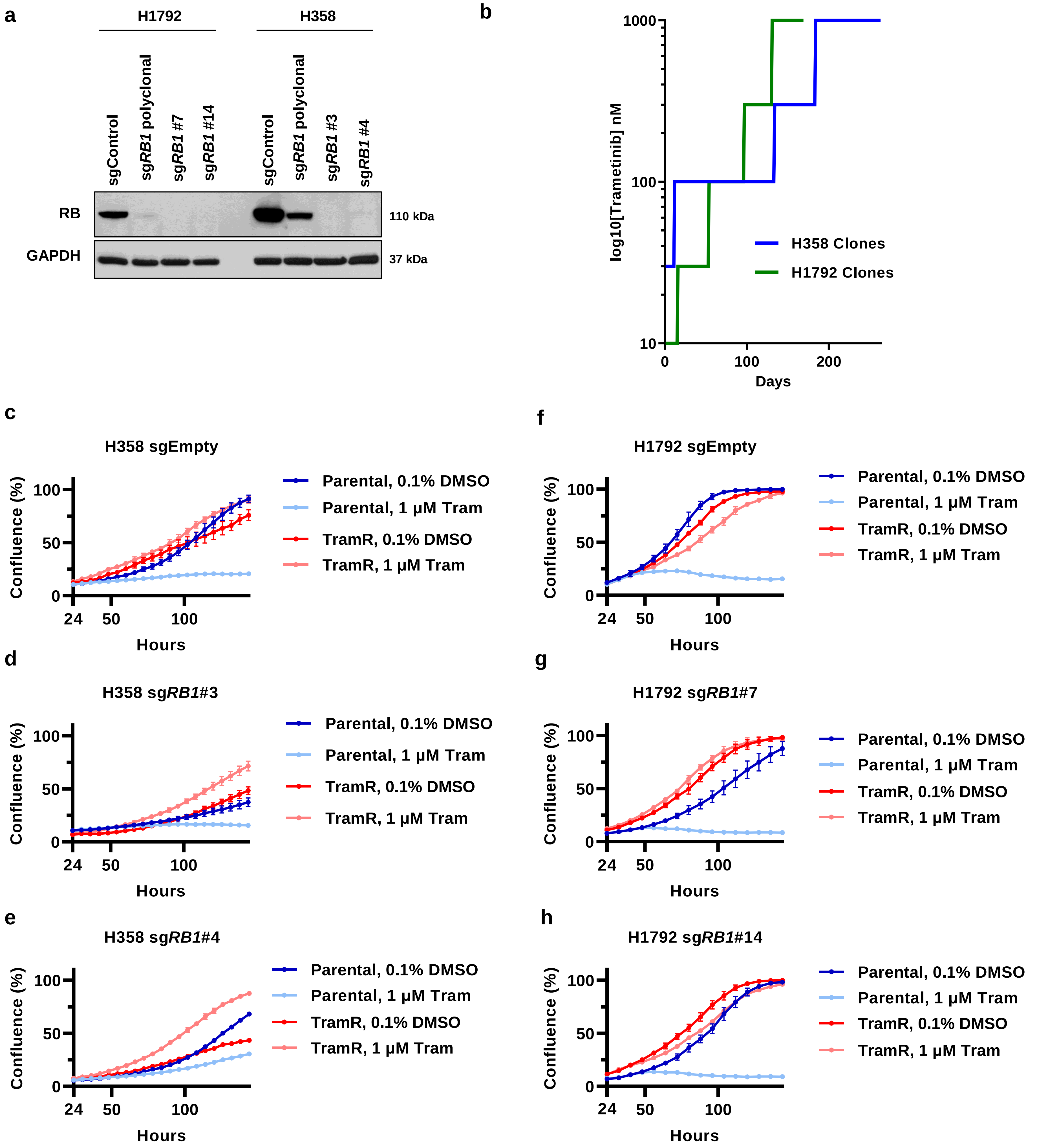

a
H1792
H358
sgControl
sgRB1 polyclonal
sgRB1 #7
sgRB1 #14
sgControl
sgRB1 polyclonal
sgRB1 #3
sgRB1 #4
RB
GAPDH
110 kDa
37 kDa
b
log10[Trametinib] nM
1000
100
10
0
100
200
Days
H358 Clones
H1792 Clones
c
H358 sgEmpty
f
H1792 sgEmpty
d
H358 sgRB1#3
g
H1792 sgRB1#7
e
H358 sgRB1#4
h
H1792 sgRB1#14
Confluence (%)
Hours
Parental, 0.1% DMSO
Parental, 1 μM Tram
TramR, 0.1% DMSO
TramR, 1 μM Tram

i

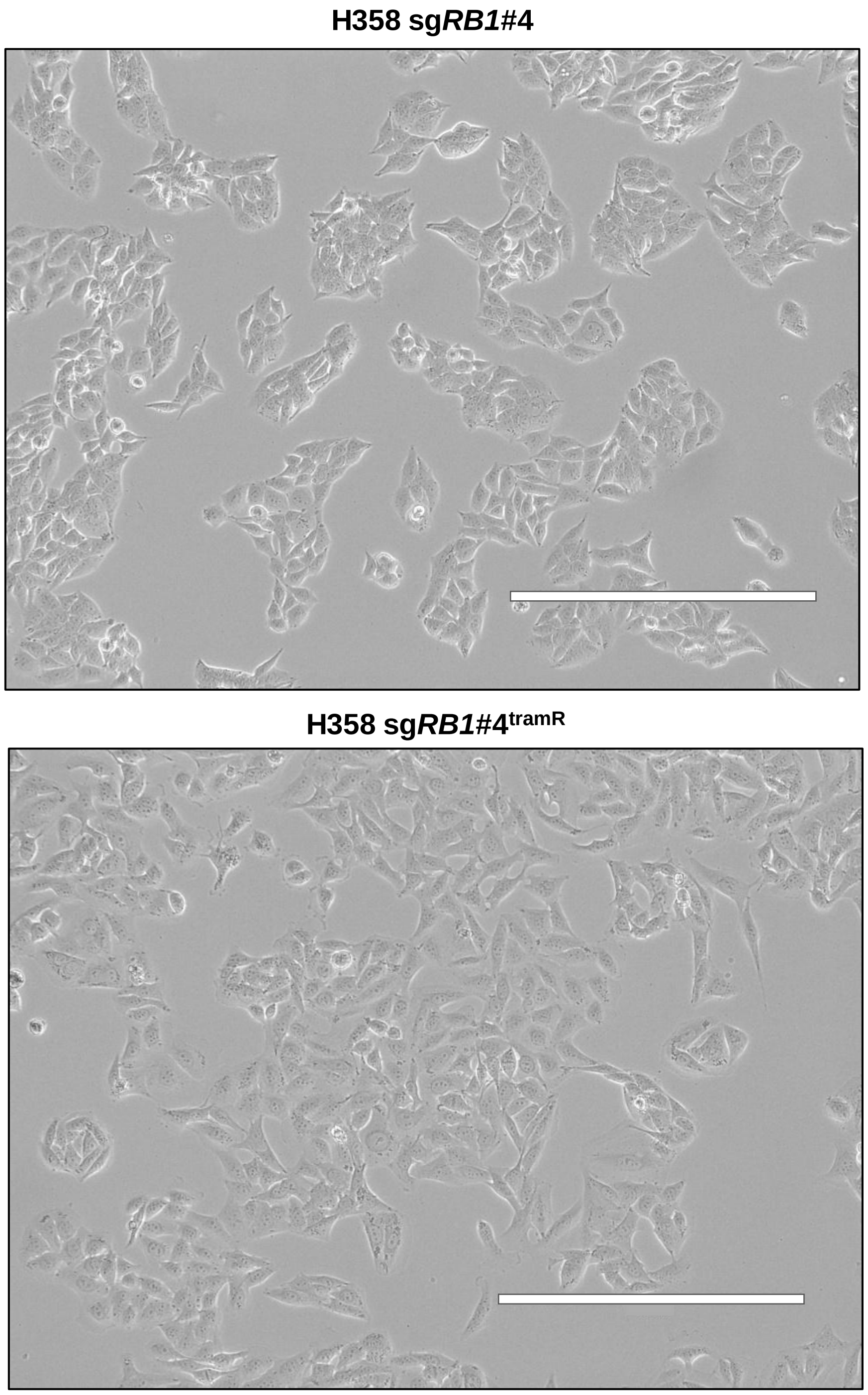

j

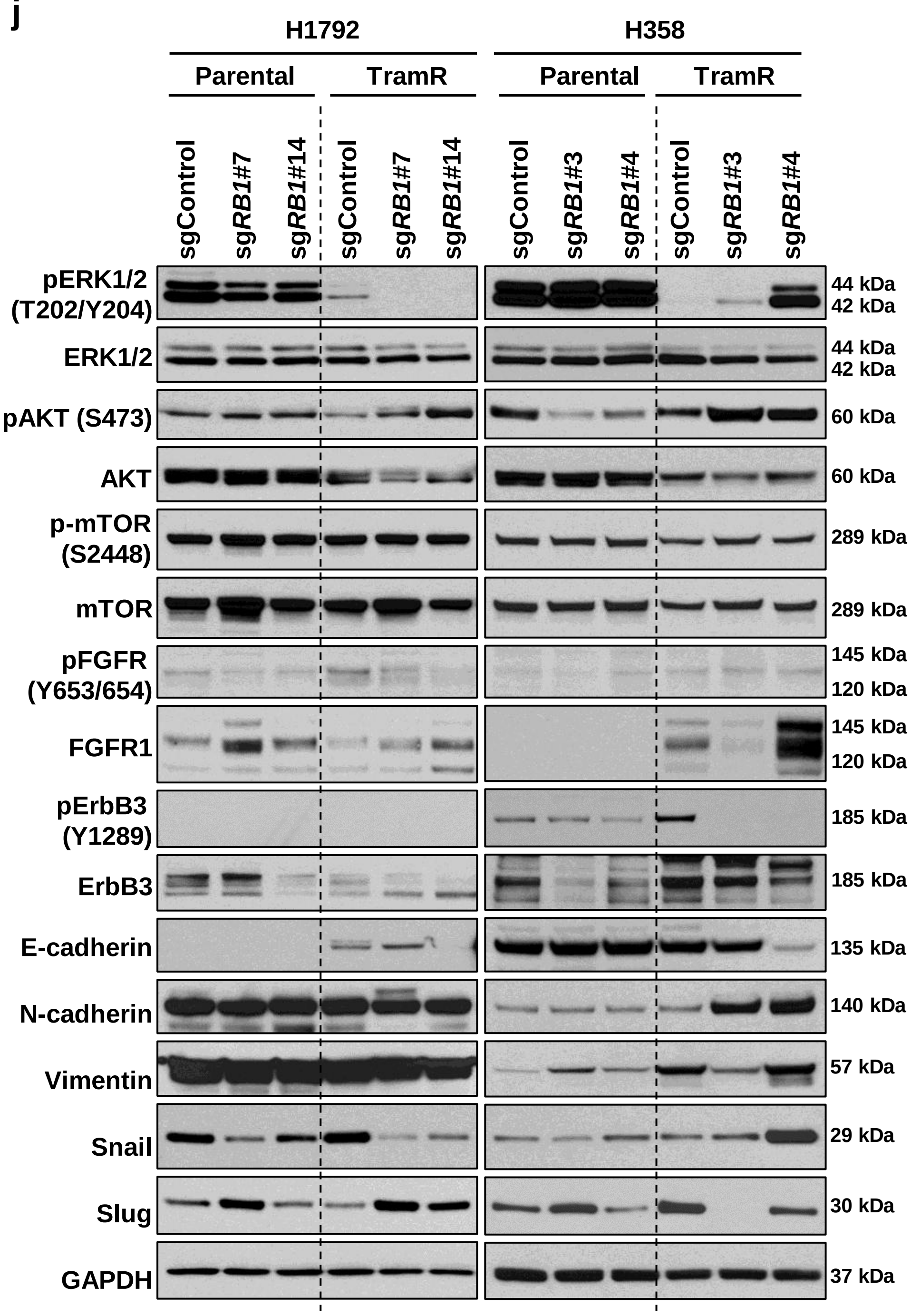

Supplementary Figure 1 Generation of trametinib resistant clones. a After transfection with sgRNA targeting RB1, polyclonal and monoclonal populations H1792 and H358 were established. Cells were grown in complete media, harvested and immunoblotted. Only monoclonal populations of H358 and H1792 displayed full RB knockout. b H358 and H1792 cells were made resistant to trametinib through culture in dose escalating concentrations of trametinib for ~200 days. Displayed are trametinib doses with respect to time in which clones were cultured during the dose escalation study. c, d, e, f, g, h Parental and resistant H1792 and H358 clones were grown in 1 µM trametinib and 0.1% DMSO, confluence was measured with IncuCyte S3 live-cell imaging system. Representative experiment from 2 independent experiments, error bars are SEM from 3 technical replicates. i 10X microscope images of parental and resistant H358 sgRB1#4 cultured in 0.1% DMSO and 1 µM trametinib, respectively. Scale bar shown represents 400 µm. j H1792 and H358 parental and resistant cells were cultured in either 0.1% DMSO (parental) or 1 µM trametinib (resistant), harvested and immunoblotted for indicated proteins. Resistant cell lines show upregulation of pathways previously associated with trametinib resistance, including PI3K/AKT, FGFR1 and ErbB3.

a

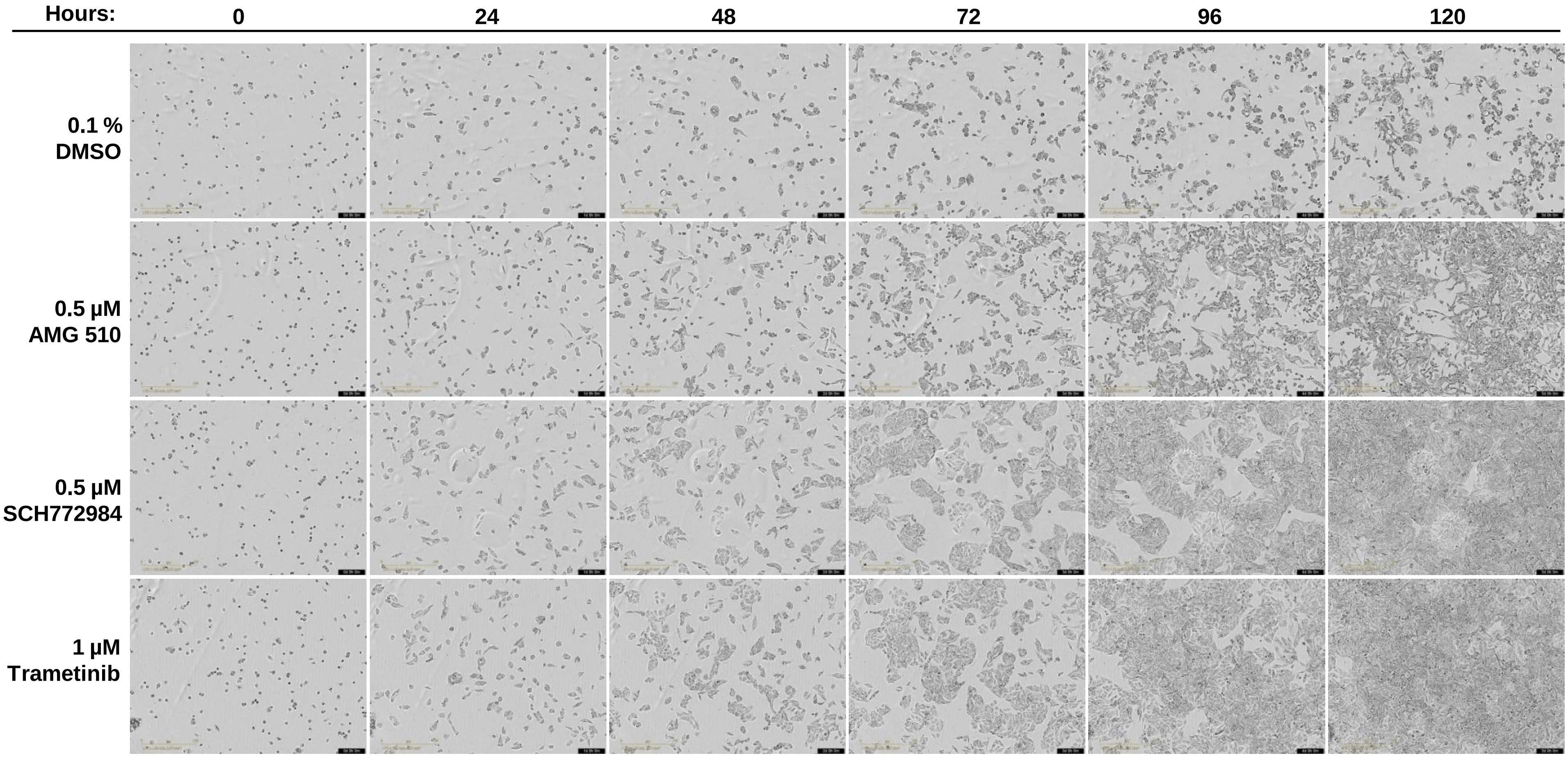

b

STR Probes	NCI-H358 (from Cellosaurus)	H358 sg*RB1*#4	H358 sg*RB1* #4[tramR]
AEML	X, Y	X, Y	X, Y
CSF1PO	11, 12	11, 12	11, 12
D13S317	8, 12	8, 12	8, 12
D16S539	12, 13	12, 13	12, 13
D5S818	10, 12	10, 12	10, 12
D7S820	10, 11	10, 11	10, 11
THO1	6	6	6
TPOX	8,9	8,9	8,9
vWA	17	17	17
D21S11	28, 30	28, 30	28, 30
D18S51	14	14	14
Penta E	18	18	18
Penta D	10, 13	10, 13	10, 13
D8S1179	13, 14	13, 14	13, 14
FGA	20, 21	20, 21	20, 21

c

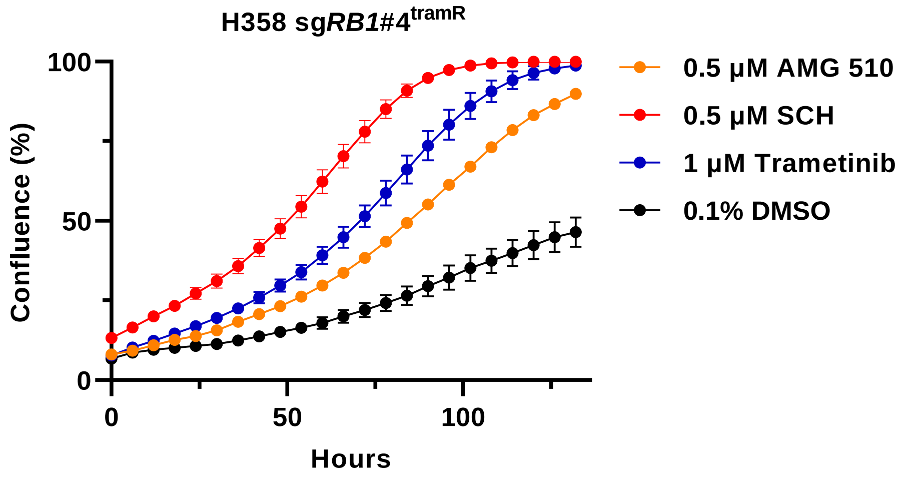

d

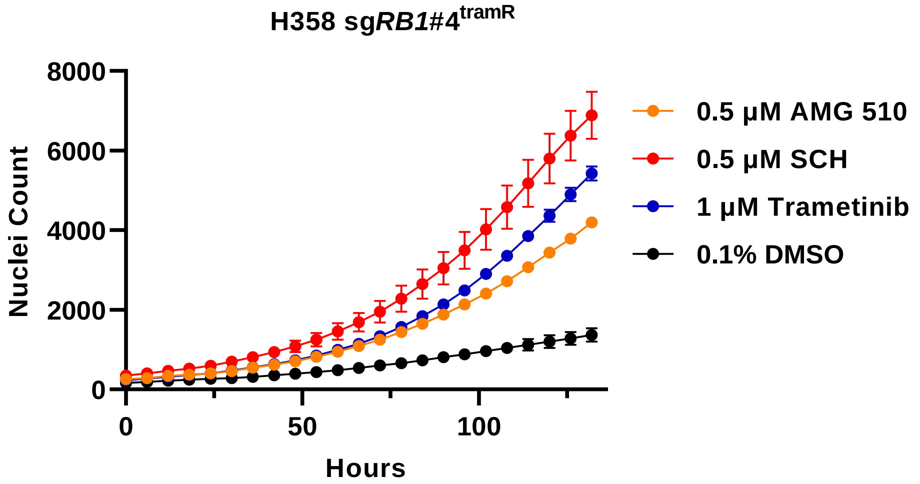

Supplementary Figure 2 H358 sgRB1#4^{tramR} lose proliferative ability following trametinib removal. a Brightfield Images taken from IncuCyte experiments on H358 sgRB1#4^{tramR}. b STR profiling on H358 sgRB1#4 parental and resistant cells, with data from Cellosaurus as reference. c, d Cell proliferation from IncuCyte proliferation assay performed on H358 sgRB1#4^{tramR} where images were collected directly following plate seeding, instead of waiting a day. Cells were treated with a nuclear marker allowing for nuclei counts. Cell proliferation is shown by cell area coverage of each image (% confluence) or by nuclei count. Images were taken from 0 hours to 144 hours. Error bars are SEM from 4 technical replicates.

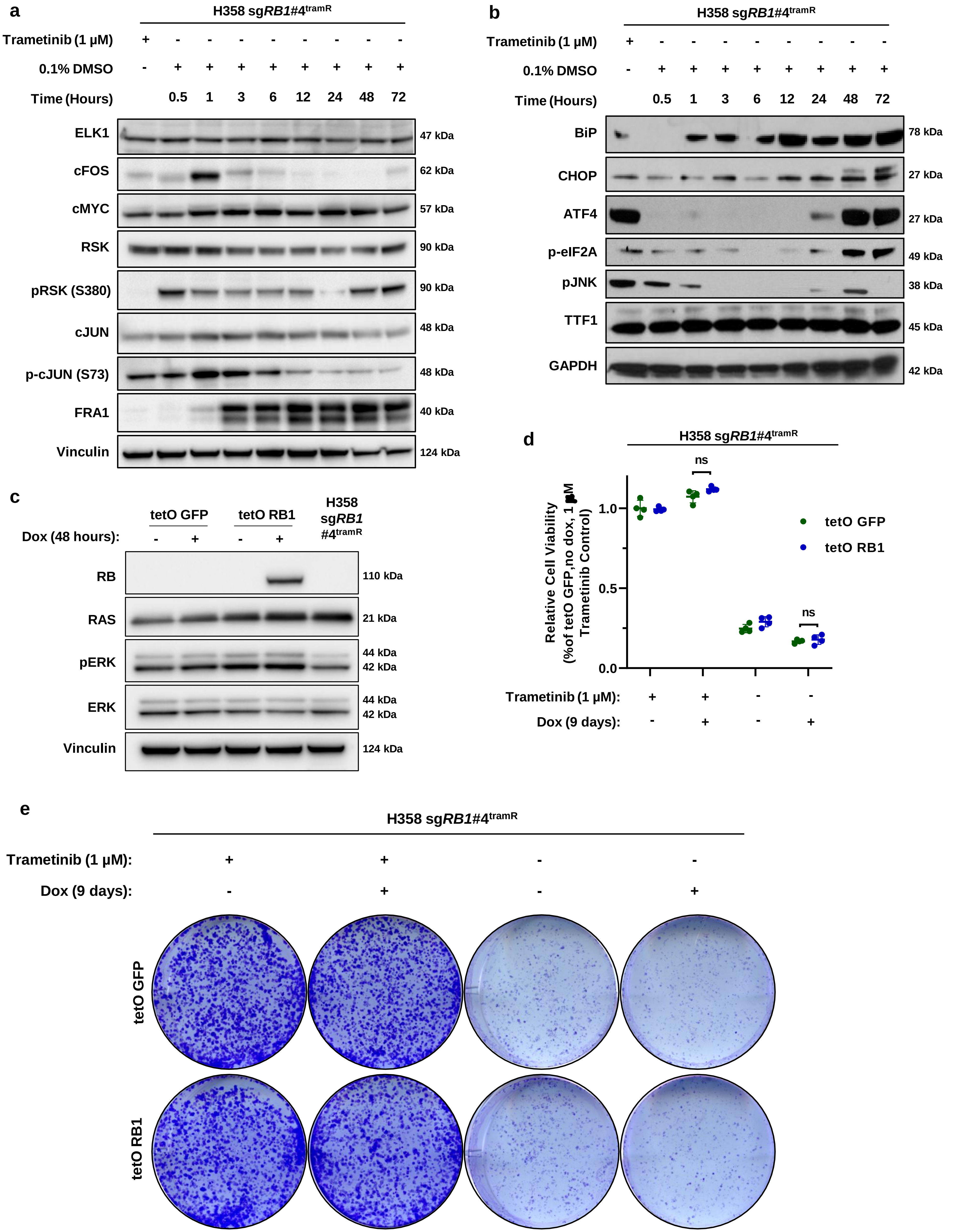
a
H358 sgRB1#4tramR
Trametinib (1 µM)
0.1% DMSO
Time (Hours)
0.5 1 3 6 12 24 48 72
ELK1 47 kDa
cFOS 62 kDa
cMYC 57 kDa
RSK 90 kDa
pRSK (S380) 90 kDa
cJUN 48 kDa
p-cJUN (S73) 48 kDa
FRA1 40 kDa
Vinculin 124 kDa
b
H358 sgRB1#4tramR
Trametinib (1 µM)
0.1% DMSO
Time (Hours)
0.5 1 3 6 12 24 48 72
BiP 78 kDa
CHOP 27 kDa
ATF4 27 kDa
p-eIF2A 49 kDa
pJNK 38 kDa
TTF1 45 kDa
GAPDH 42 kDa
c
tetO GFP
tetO RB1
H358 sgRB1 #4tramR
Dox (48 hours):
RB 110 kDa
RAS 21 kDa
pERK 44 kDa 42 kDa
ERK 44 kDa 42 kDa
Vinculin 124 kDa
d
H358 sgRB1#4tramR
Relative Cell Viability (%of tetO GFP,no dox, 1 µM Trametinib Control)
ns
ns
1.0
0.5
0.0
tetO GFP
tetO RB1
Trametinib (1 µM):
Dox (9 days):
e
H358 sgRB1#4tramR
Trametinib (1 µM):
Dox (9 days):
tetO GFP
tetO RB1

Supplementary Figure 3 Upregulation of ER stress associated proteins precedes apoptosis after drug removal in H358 sgRB1#4^{tramR}. a H358 sgRB1#4^{tramR} were treated with 0.1% DMSO or 1 µM trametinib, harvested after indicated time periods and immunoblotted for indicated proteins. ERK1/2 substrates are upregulated or phosphorylated after trametinib removal. b ER stress signaling proteins are present while the cells are grown in 1 µM trametinib and following drug removal. c H358 sgRB1#4^{tramR} tetO GFP and RB1 cells were treated 100ng/ml dox for 48 hours, harvested and immunoblotted with lysate from non-transfected H358sgRB1#4^{tramR} cells as a control. d, e H358 sgRB1#4^{tramR} tetO GFP and RB1 cells were treated with 100ng/ml and 1 µM trametinib or 0.1% DMSO for 9 days, treated with alamarBlue at endpoint and stained with crystal violet. All values are relative to H358 sgRB1#4^{tramR} tetO GFP treated with 1 µM trametinib, no dox. P value from students t test on relative viabilities shown, ns = not significant. Error bars represent SD from 4 independent replicates. Crystal violet staining images shown are representative from 4 independent replicates.

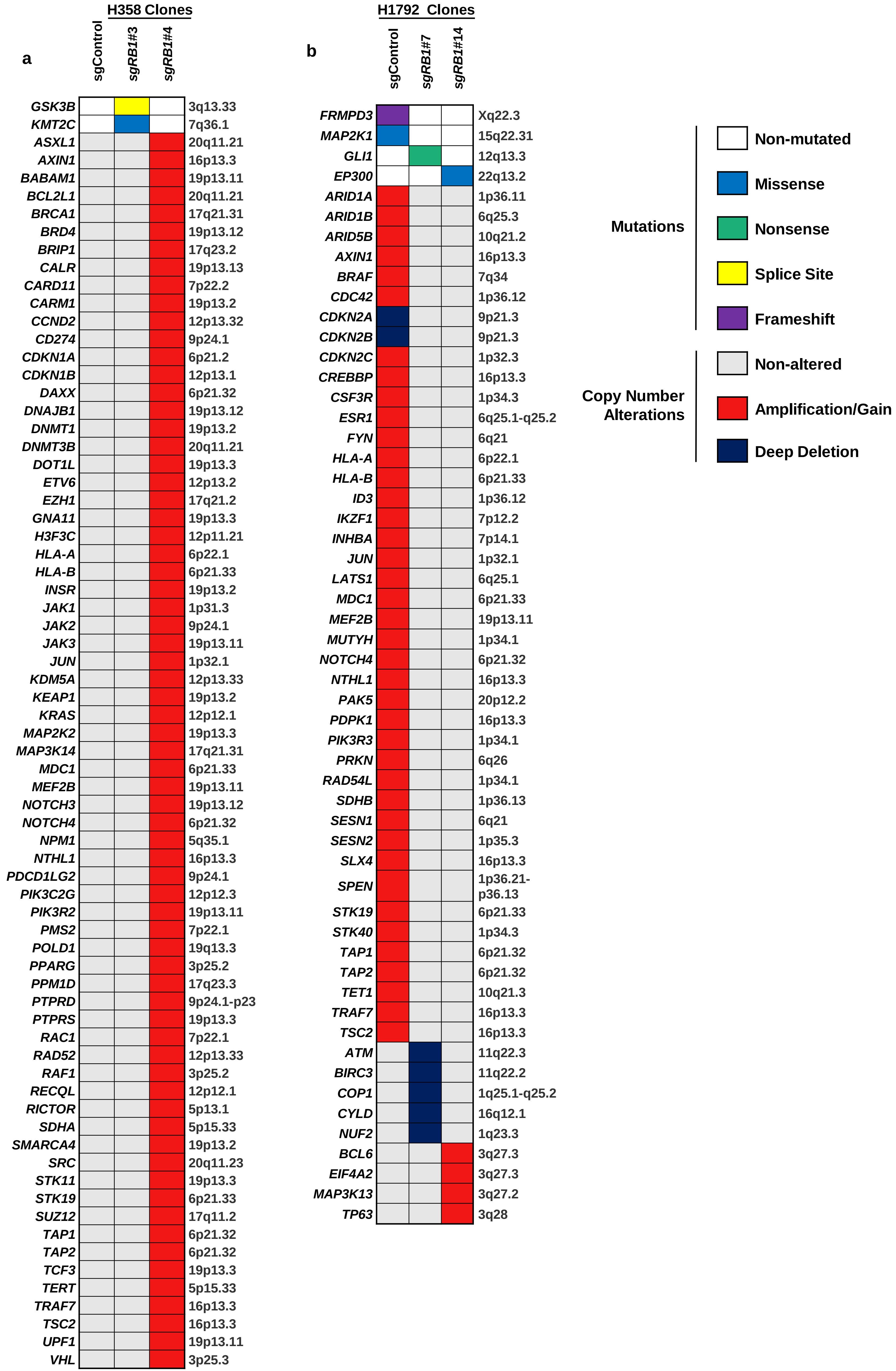
a
H358 Clones
sgControl
sgRB1#3
sgRB1#4
GSK3B 3q13.33
KMT2C 7q36.1
ASXL1 20q11.21
AXIN1 16p13.3
BABAM1 19p13.11
BCL2L1 20q11.21
BRCA1 17q21.31
BRD4 19p13.12
BRIP1 17q23.2
CALR 19p13.13
CARD11 7p22.2
CARM1 19p13.2
CCND2 12p13.32
CD274 9p24.1
CDKN1A 6p21.2
CDKN1B 12p13.1
DAXX 6p21.32
DNAJB1 19p13.12
DNMT1 19p13.2
DNMT3B 20q11.21
DOT1L 19p13.3
ETV6 12p13.2
EZH1 17q21.2
GNA11 19p13.3
H3F3C 12p11.21
HLA-A 6p22.1
HLA-B 6p21.33
INSR 19p13.2
JAK1 1p31.3
JAK2 9p24.1
JAK3 19p13.11
JUN 1p32.1
KDM5A 12p13.33
KEAP1 19p13.2
KRAS 12p12.1
MAP2K2 19p13.3
MAP3K14 17q21.31
MDC1 6p21.33
MEF2B 19p13.11
NOTCH3 19p13.12
NOTCH4 6p21.32
NPM1 5q35.1
NTHL1 16p13.3
PDCD1LG2 9p24.1
PIK3C2G 12p12.3
PIK3R2 19p13.11
PMS2 7p22.1
POLD1 19q13.3
PPARG 3p25.2
PPM1D 17q23.3
PTPRD 9p24.1-p23
PTPRS 19p13.3
RAC1 7p22.1
RAD52 12p13.33
RAF1 3p25.2
RECQL 12p12.1
RICTOR 5p13.1
SDHA 5p15.33
SMARCA4 19p13.2
SRC 20q11.23
STK11 19p13.3
STK19 6p21.33
SUZ12 17q11.2
TAP1 6p21.32
TAP2 6p21.32
TCF3 19p13.3
TERT 5p15.33
TRAF7 16p13.3
TSC2 16p13.3
UPF1 19p13.11
VHL 3p25.3
b
H1792 Clones
sgControl
sgRB1#7
sgRB1#14
FRMPD3 Xq22.3
MAP2K1 15q22.31
GLI1 12q13.3
EP300 22q13.2
ARID1A 1p36.11
ARID1B 6q25.3
ARID5B 10q21.2
AXIN1 16p13.3
BRAF 7q34
CDC42 1p36.12
CDKN2A 9p21.3
CDKN2B 9p21.3
CDKN2C 1p32.3
CREBBP 16p13.3
CSF3R 1p34.3
ESR1 6q25.1-q25.2
FYN 6q21
HLA-A 6p22.1
HLA-B 6p21.33
ID3 1p36.12
IKZF1 7p12.2
INHBA 7p14.1
JUN 1p32.1
LATS1 6q25.1
MDC1 6p21.33
MEF2B 19p13.11
MUTYH 1p34.1
NOTCH4 6p21.32
NTHL1 16p13.3
PAK5 20p12.2
PDPK1 16p13.3
PIK3R3 1p34.1
PRKN 6q26
RAD54L 1p34.1
SDHB 1p36.13
SESN1 6q21
SESN2 1p35.3
SLX4 16p13.3
SPEN 1p36.21-p36.13
STK19 6p21.33
STK40 1p34.3
TAP1 6p21.32
TAP2 6p21.32
TET1 10q21.3
TRAF7 16p13.3
TSC2 16p13.3
ATM 11q22.3
BIRC3 11q22.2
COP1 1q25.1-q25.2
CYLD 16q12.1
NUF2 1q23.3
BCL6 3q27.3
EIF4A2 3q27.3
MAP3K13 3q27.2
TP63 3q28
Mutations
Non-mutated
Missense
Nonsense
Splice Site
Frameshift
Copy Number Alterations
Non-altered
Amplification/Gain
Deep Deletion

Supplementary Figure 4 MSK-IMPACT sequencing offers insight into potential mechanisms of adaptive resistance to trametinib. a Trametinib resistant H358 and b H1792 RB knockout and control cells were submitted for MSK-IMPACT sequencing, a targeted panel which screens for actionable mutations. Mutations and copy number alterations for each sample were determined by comparing the results to parental version of the cell. Chromosomal location of each gene is displayed.

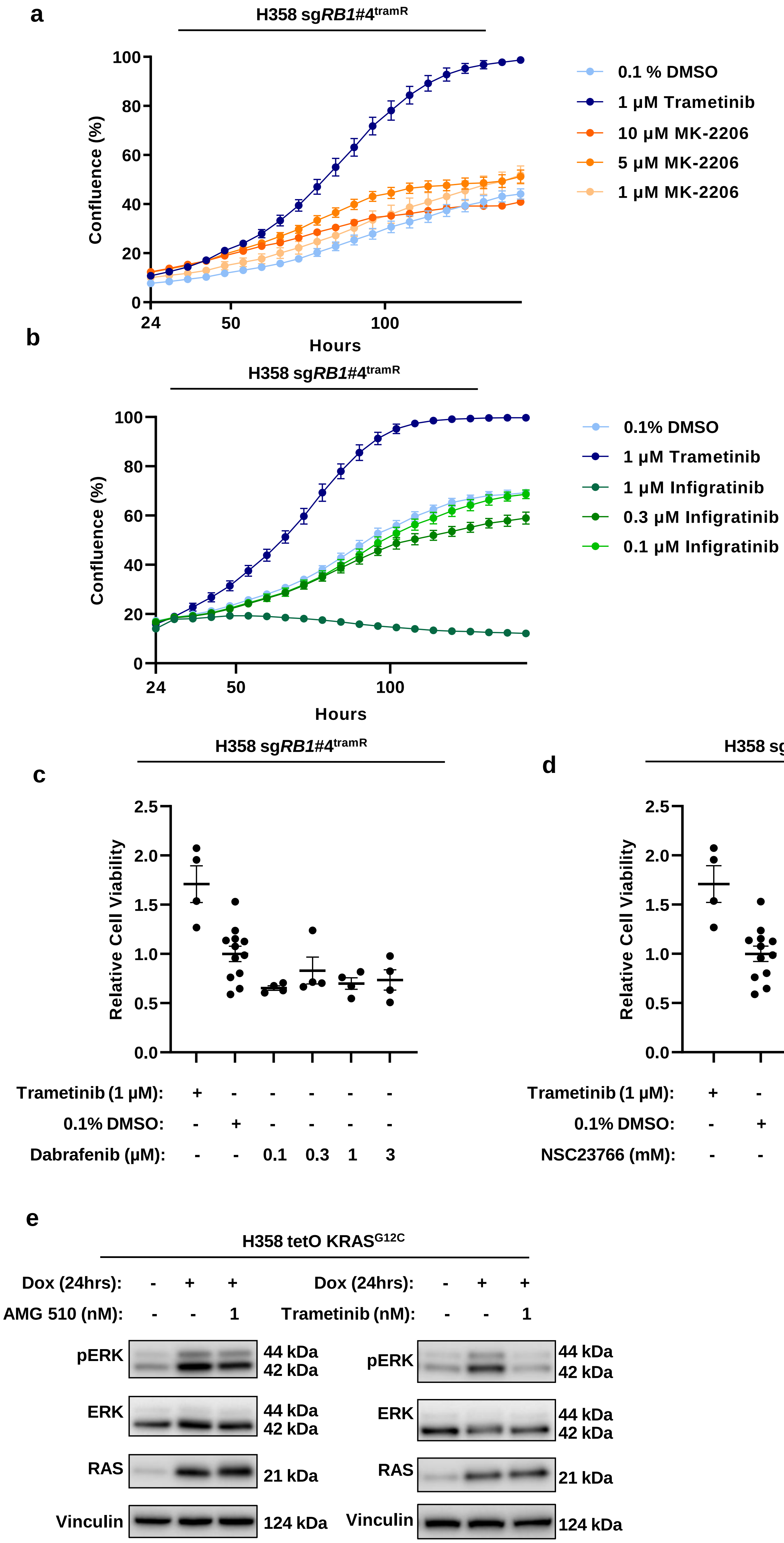

a
H358 sg*RB1#4*tramR
Confluence (%)
0
20
40
60
80
100
24
50
100
Hours
0.1 % DMSO
1 µM Trametinib
10 µM MK-2206
5 µM MK-2206
1 µM MK-2206
b
H358 sg*RB1#4*tramR
Confluence (%)
0
20
40
60
80
100
24
50
100
Hours
0.1% DMSO
1 µM Trametinib
1 µM Infigratinib
0.3 µM Infigratinib
0.1 µM Infigratinib
c
H358 sg*RB1#4*tramR
Relative Cell Viability
0.0
0.5
1.0
1.5
2.0
2.5
Trametinib (1 µM): + - - - - -
0.1% DMSO: - + - - - -
Dabrafenib (µM): - - 0.1 0.3 1 3
d
H358 sg*RB1#4*tramR
Relative Cell Viability
0.0
0.5
1.0
1.5
2.0
2.5
Trametinib (1 µM): + - - - -
0.1% DMSO: - + - - -
NSC23766 (mM): - - 1 5 10
e
H358 tetO KRASG12C
Dox (24hrs): - + +
AMG 510 (nM): - - 1
Dox (24hrs): - + +
Trametinib (nM): - - 1
pERK
44 kDa
42 kDa
ERK
44 kDa
42 kDa
RAS
21 kDa
Vinculin
124 kDa
pERK
44 kDa
42 kDa
ERK
44 kDa
42 kDa
RAS
21 kDa
Vinculin
124 kDa

f

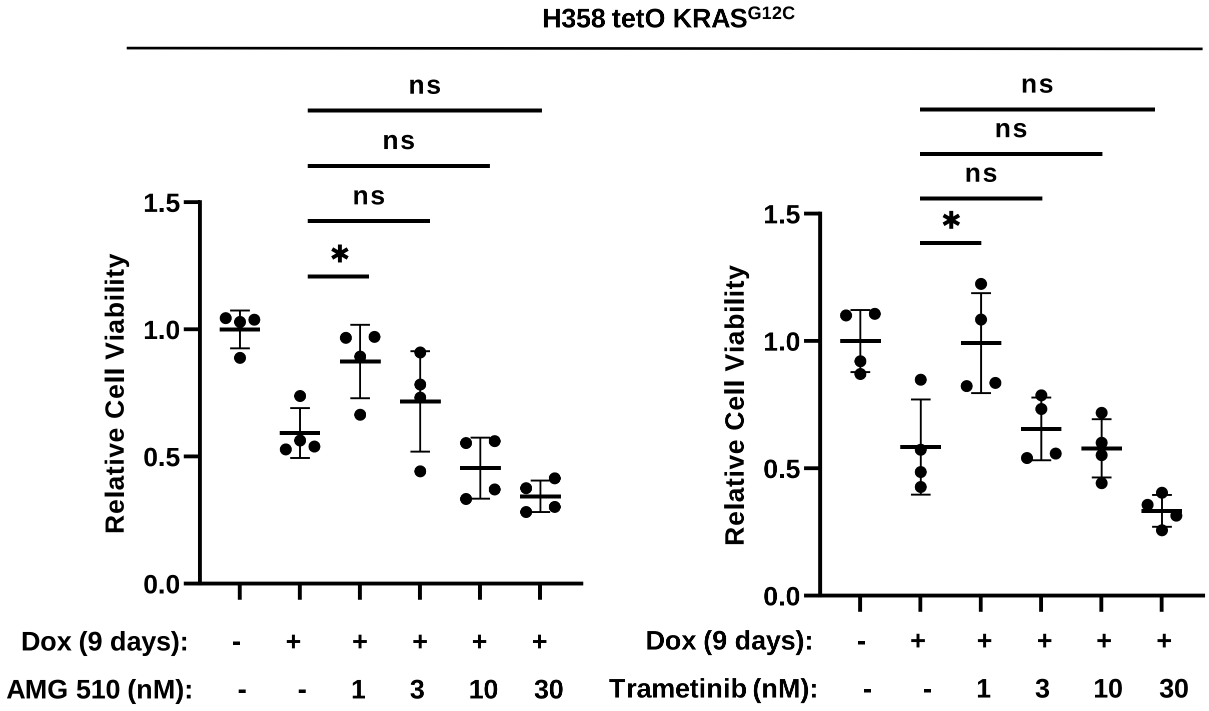
H358 tetO KRAS G12C
Relative Cell Viability
Dox (9 days): - + + + + +
AMG 510 (nM): - - 1 3 10 30
Trametinib (nM): - - 1 3 10 30
ns
*

g

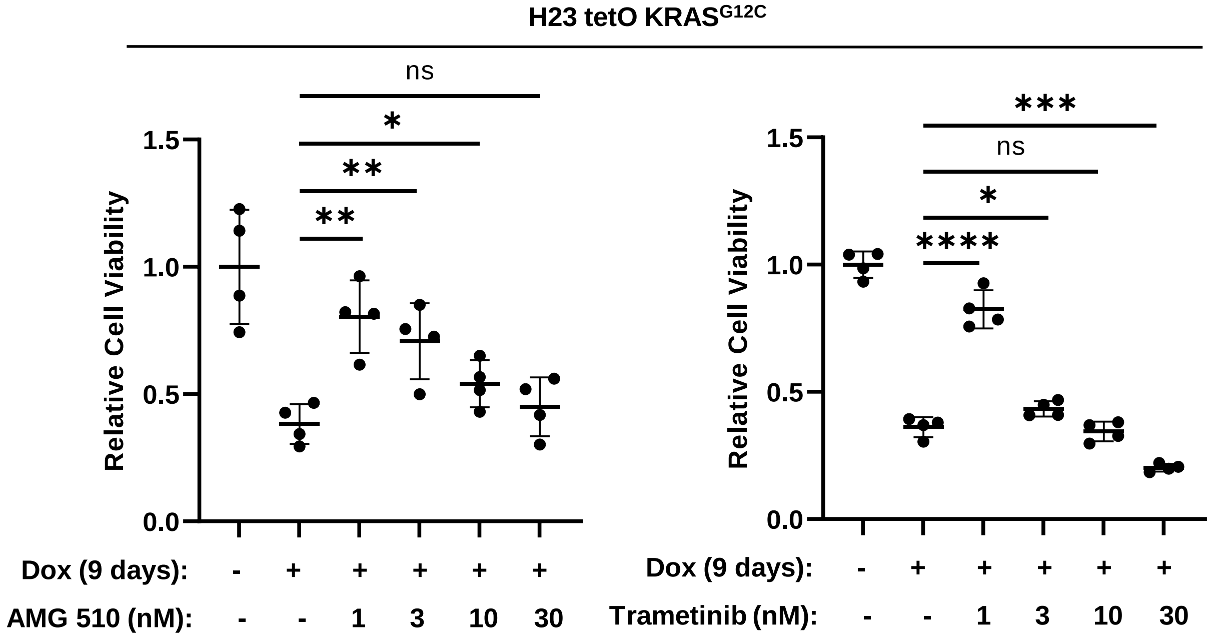
H23 tetO KRAS G12C
Relative Cell Viability
Dox (9 days): - + + + + +
AMG 510 (nM): - - 1 3 10 30
Trametinib (nM): - - 1 3 10 30
ns
*
**
***
****

h

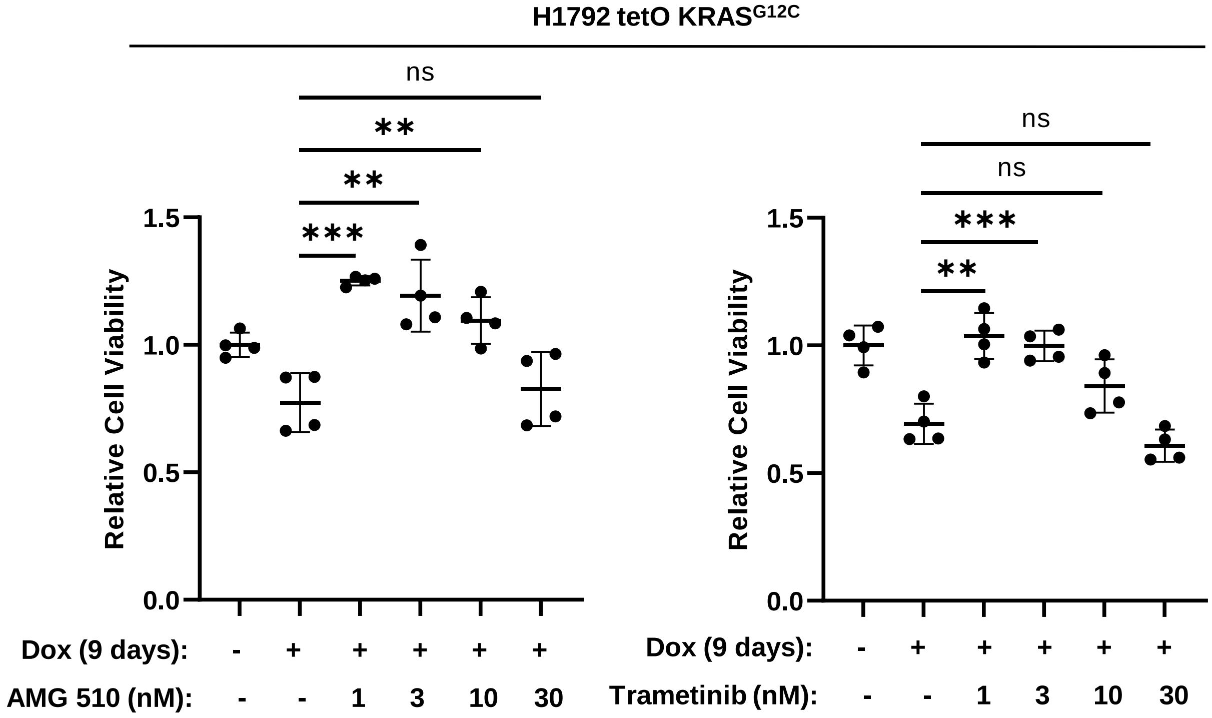
H1792 tetO KRAS G12C
Relative Cell Viability
Dox (9 days): - + + + + +
AMG 510 (nM): - - 1 3 10 30
Trametinib (nM): - - 1 3 10 30
ns
**
***

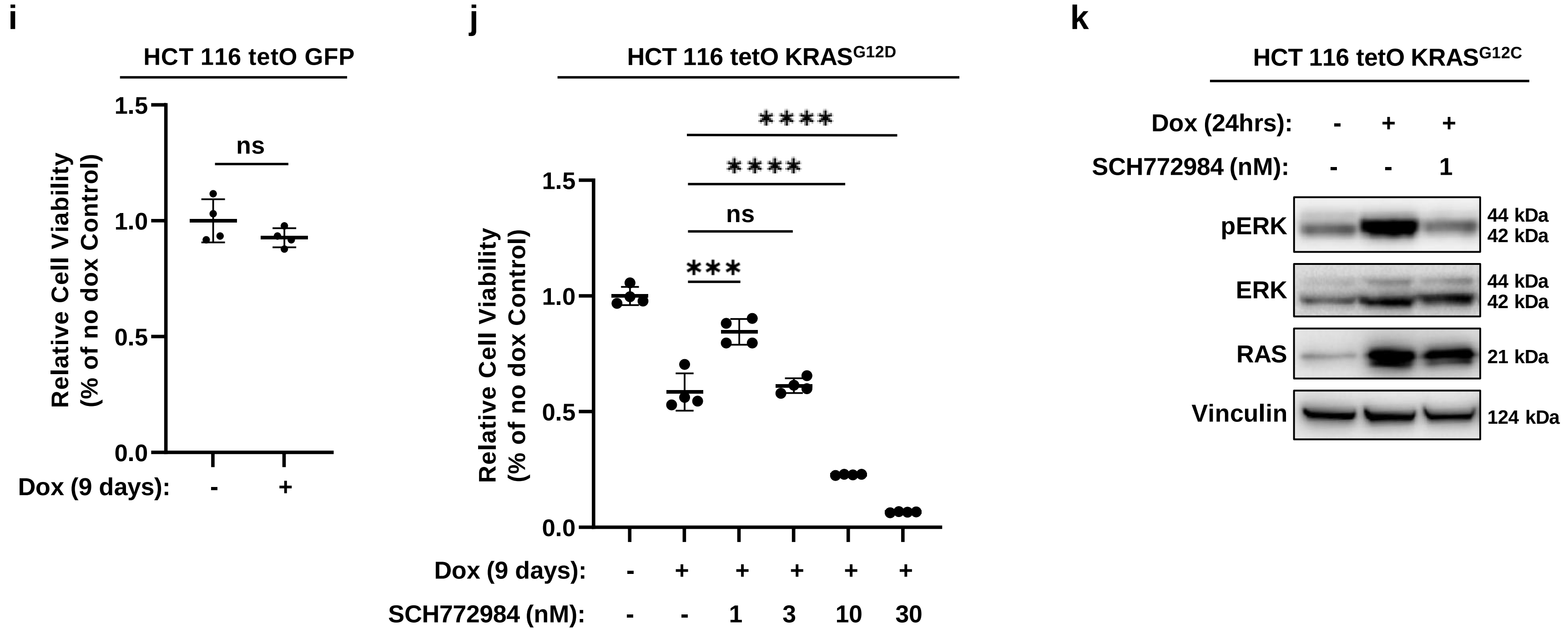

Supplementary Figure 5 Inhibition of $KRAS^{G12C}$ or ERK is the only way to rescue drug addiction following trametinib removal in H358 sgRB1#4^{tramR}. a, b H358 sgRB1#4^{tramR} displays upregulation of FGFR1 and activation of the PI3K/AKT pathway. Treatment with MK-2206, an AKT inhibitor, or Infigratinib, an FGFR1 inhibitor, does not rescue cell growth after trametinib removal, as measured by IncuCyte S3 live-cell imaging system. Error bars represent 4 technical replicates. c, d Treatment with Dabrafenib or NSC23766, inhibitors for c-RAF and Rac-1 respectively, does not rescue loss of viability after trametinib removal. Error bars represent SEM from 4 technical replicates. e H358 cell lines were made to express $KRAS^{G12C}$ by treatment with 200 ng/mL doxycycline. Inhibition of MEK or $KRAS^{G12C}$ specifically treatment with 1 nM trametinib or 1 nM AMG 510 partially rescues pERK by $KRAS^{G12C}$ after 24 hours. f, g, h H358, H23 and H1792 tetO $KRAS^{G12C}$ were treated with dox for 9 days. Increasing concentrations of AMG 510 or trametinib results in poorer rescue from the toxic effects of $KRAS^{G12C}$. Cell viability was measured by alamarBlue and calculated relative to vehicle control. Error bars represent SD from 4 independent experiments. i HCT 116 cells grown with or without 1 µg/ml of dox display no significant loss of viability. j, k Induction of $KRAS^{G12D}$ with 1 µg/ml dox in HCT 116 cells results in loss of viability after 9 days and induction of pERK after 24 hours. Both are reversed when cells are treated with 1 nM SCH772984. Cell viability was measured by alamarBlue and calculated relative to vehicle control. Error bars represent SD from 4 independent experiments. p values from student's t test are indicated. $*p < 0.05$, $**p < 0.01$, $***p < 0.001$, $****p < 0.0001$, ns = not significant.

Uncropped Figure 1a

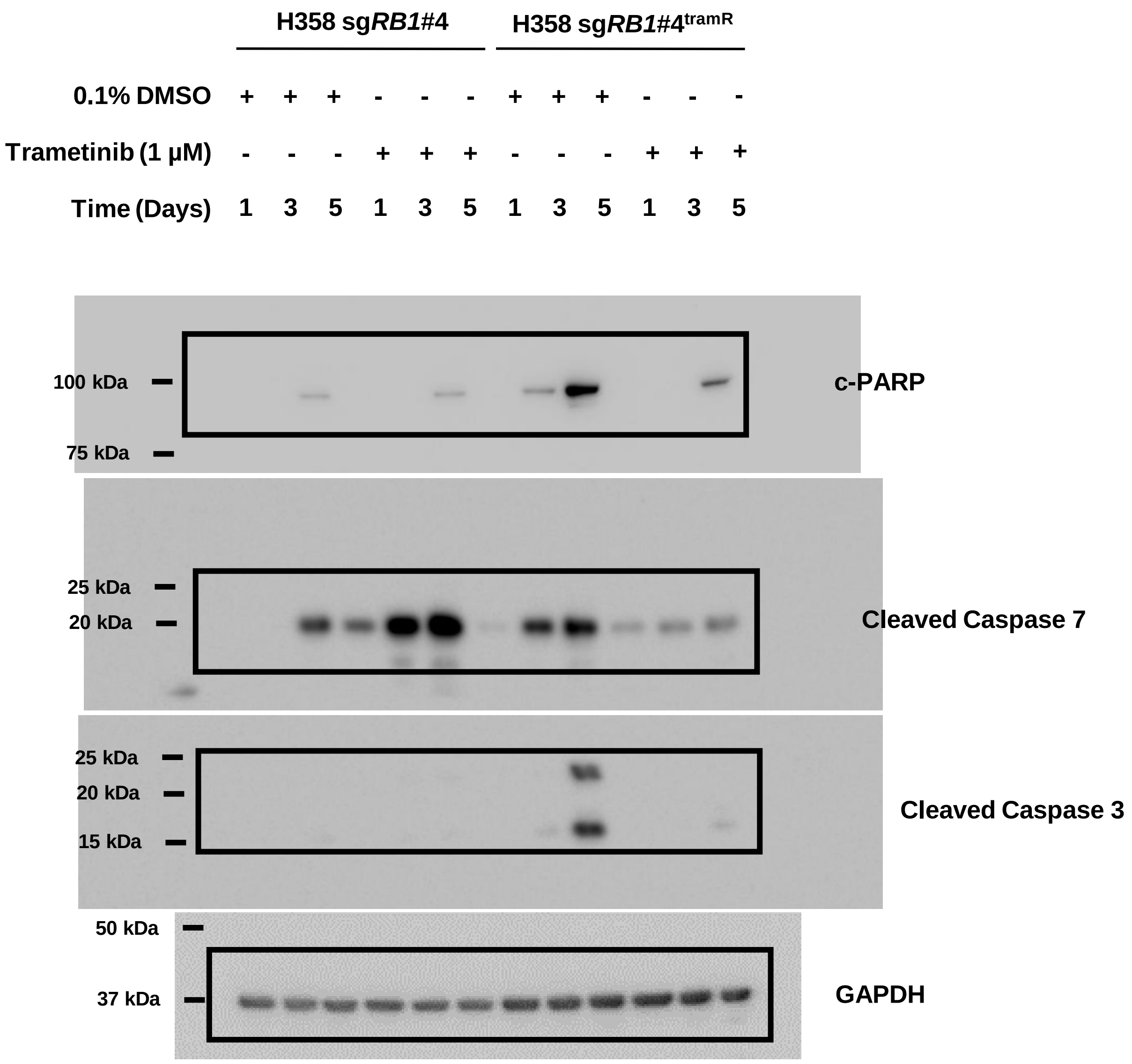

Supplementary Figure 6 Uncropped immunoblot images from indicated figure.

Uncropped Figure 3a

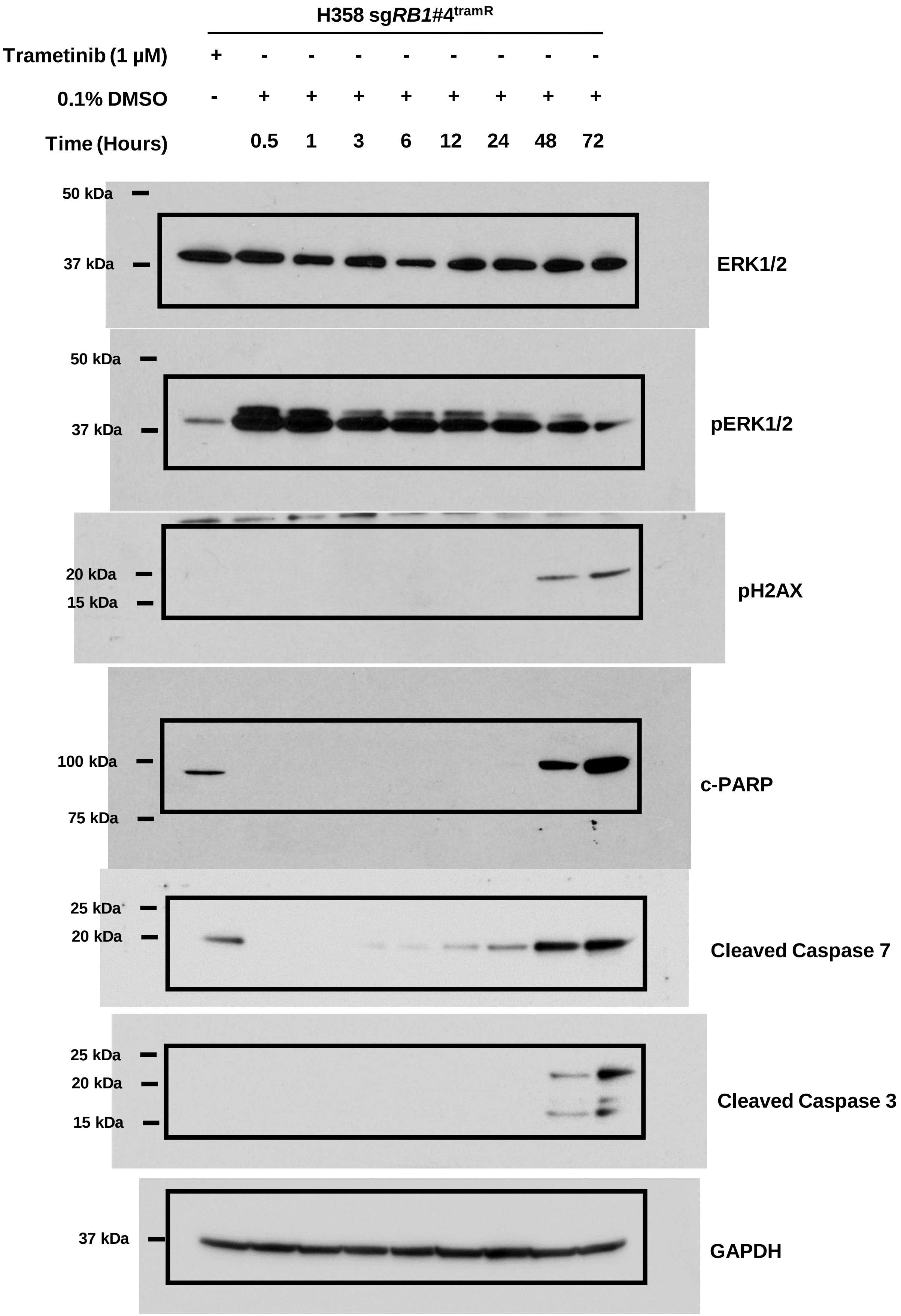

Supplementary Figure 7 Uncropped immunoblot images from indicated figure.

Uncropped Figure 3c

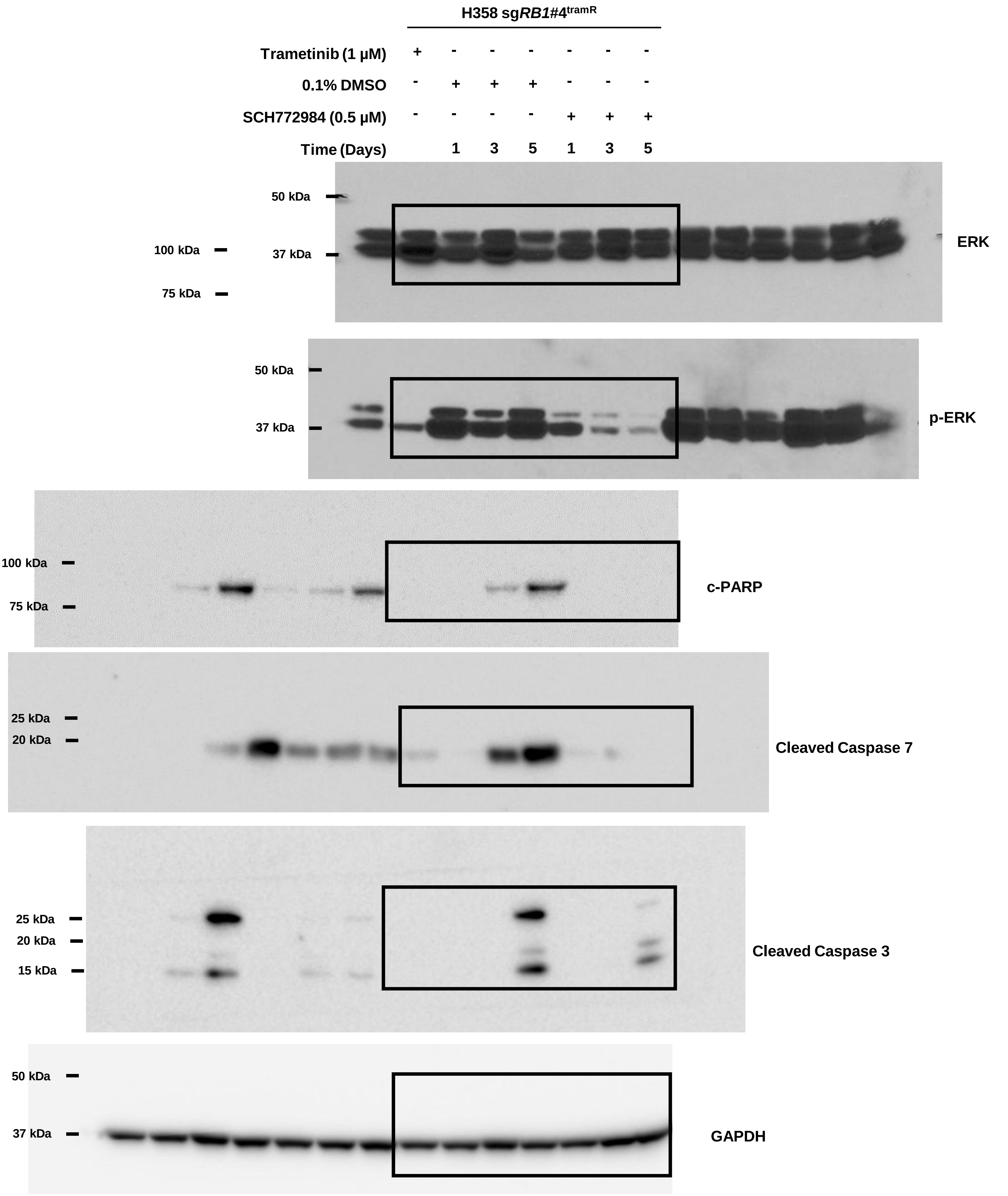

Supplementary Figure 8 Uncropped immunoblot images from indicated figure.

Uncropped Figure 3d

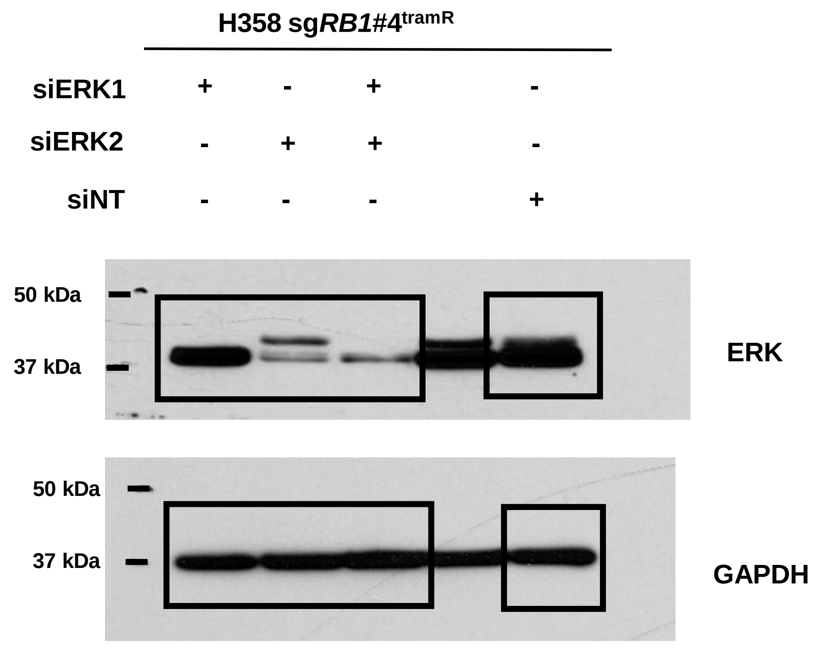

Supplementary Figure 9 Uncropped immunoblot images from indicated figure.

Uncropped Figure 4b

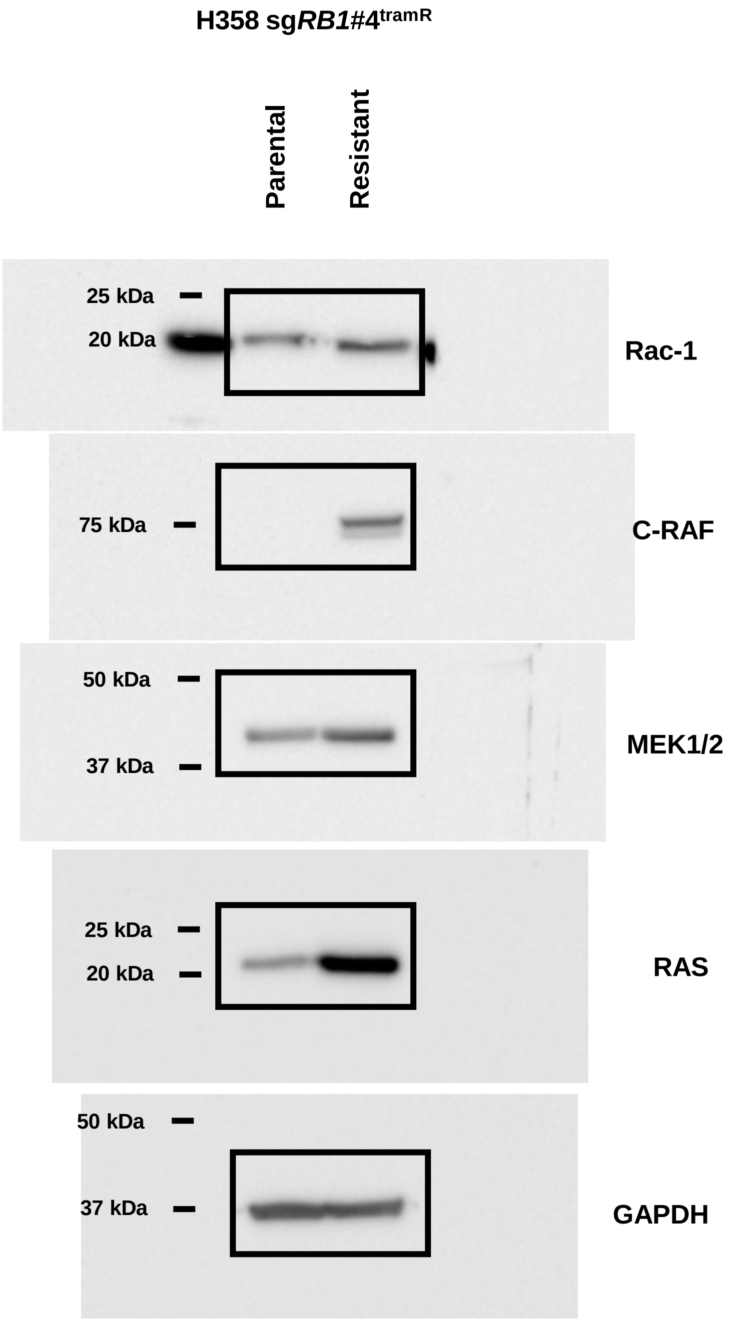

Supplementary Figure 10 Uncropped immunoblot images from indicated figure.

Uncropped Figure 5a

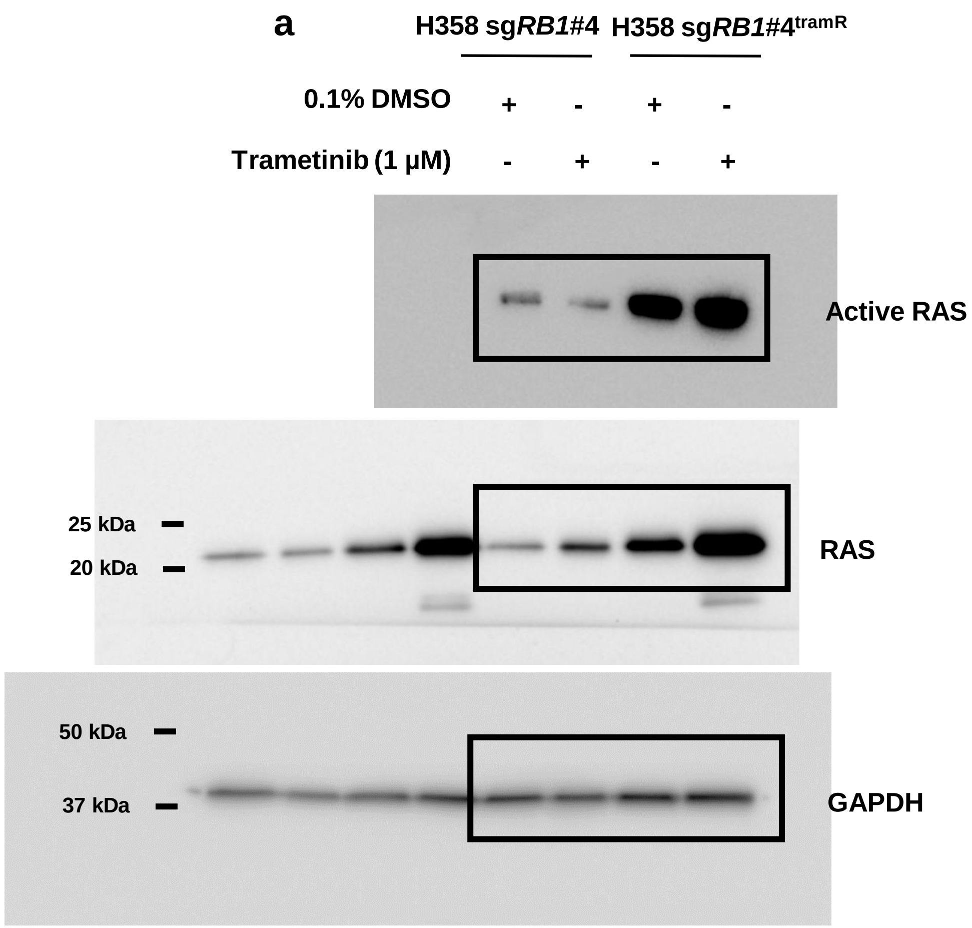

Supplementary Figure 11 Uncropped immunoblot images from indicated figure.

Uncropped Figure 5b

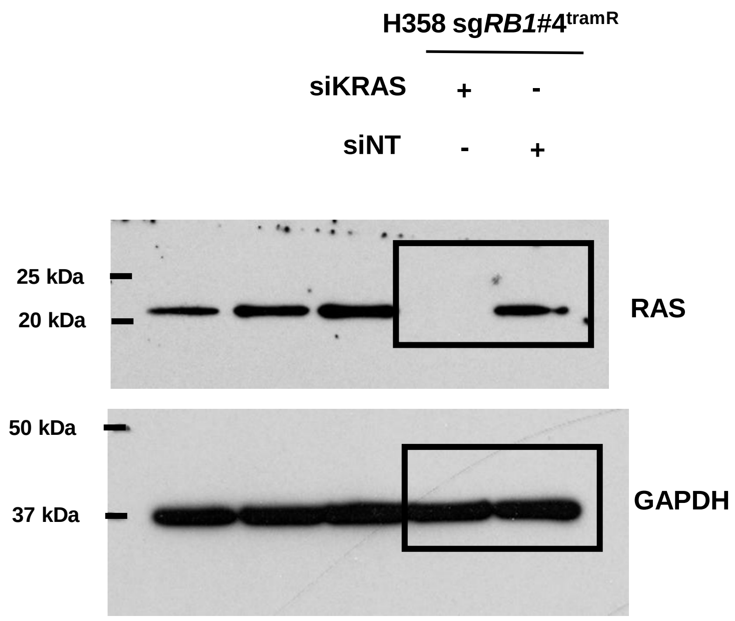

Supplementary Figure 12 Uncropped immunoblot images from indicated figure.

Uncropped Figure 5e

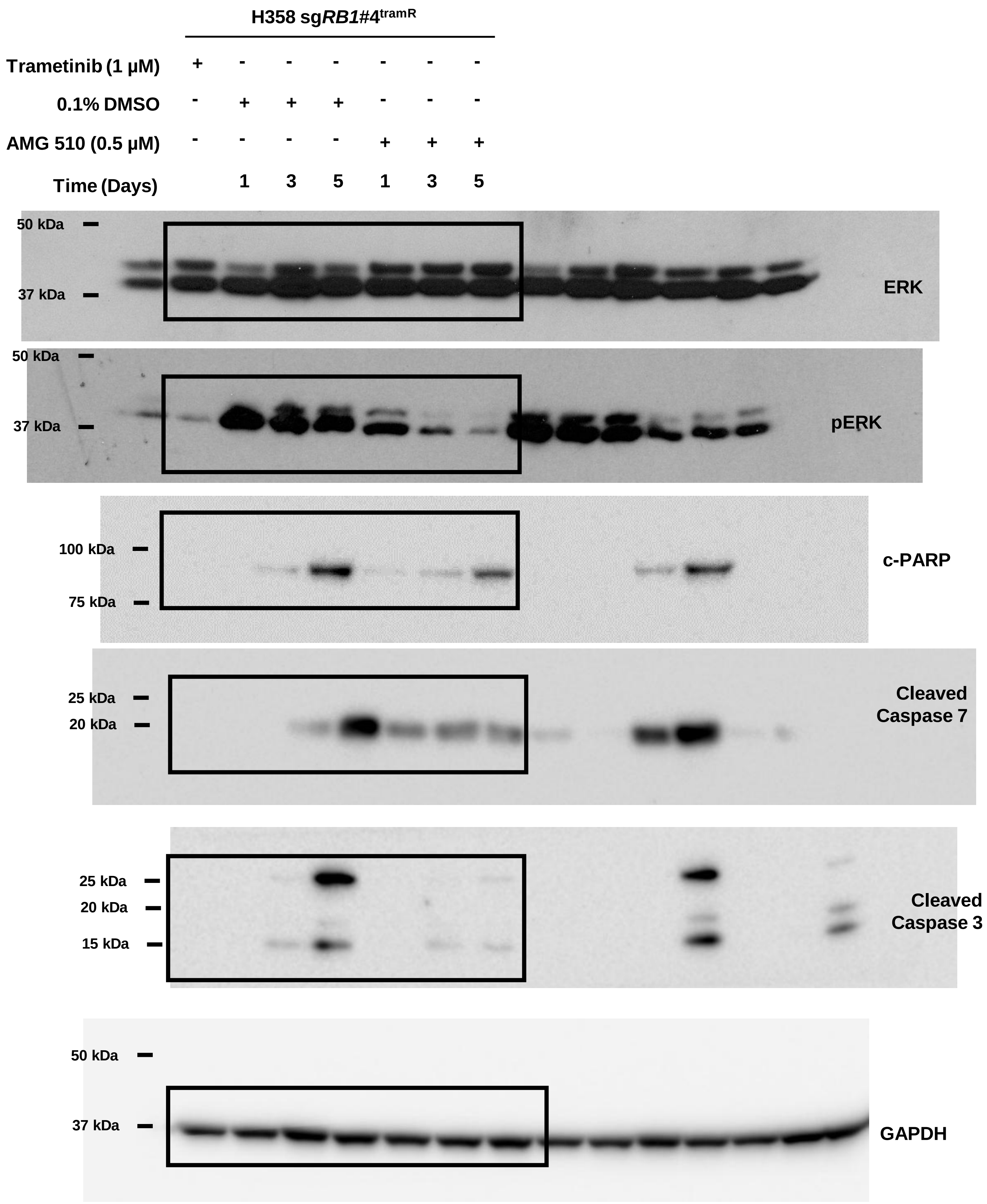

Supplementary Figure 13 Uncropped immunoblot images from indicated figure.

Uncropped Figure 5f

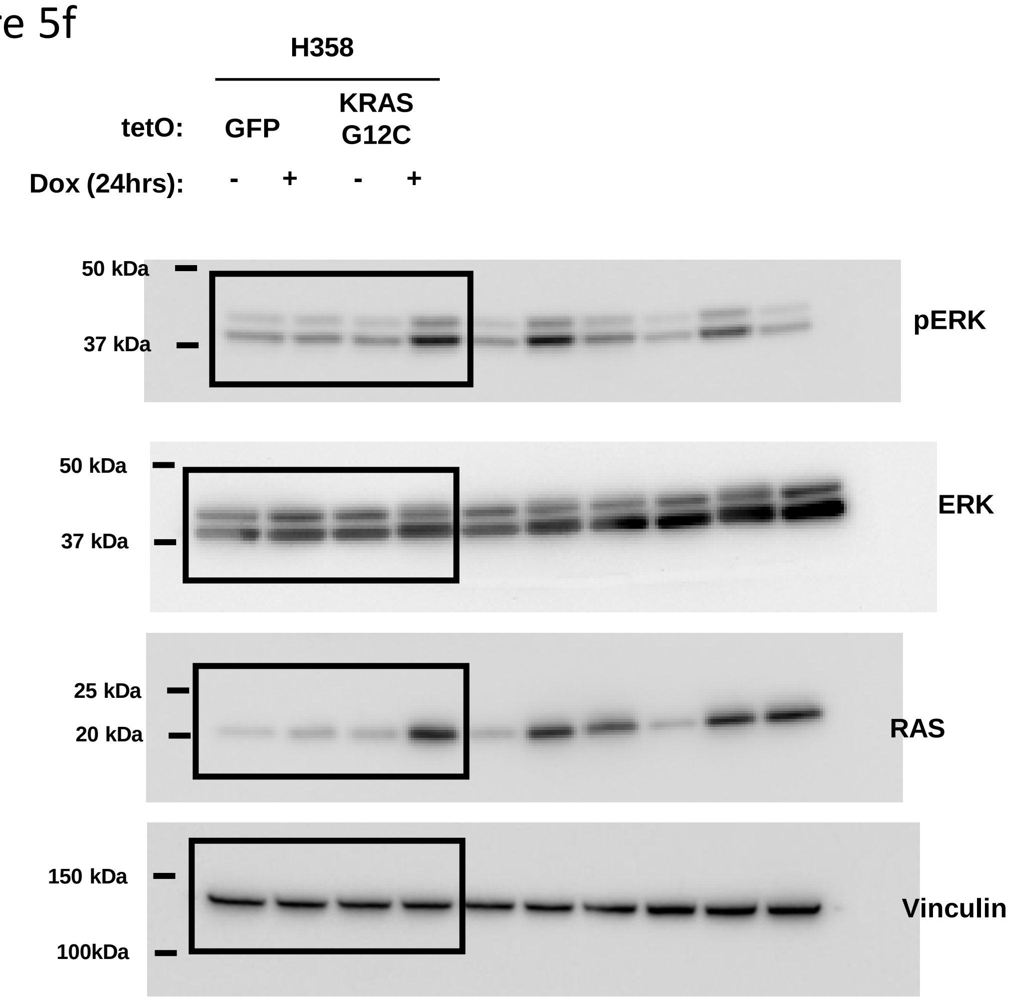

Supplementary Figure 14 Uncropped immunoblot images from indicated figure.

Uncropped Figure 5g

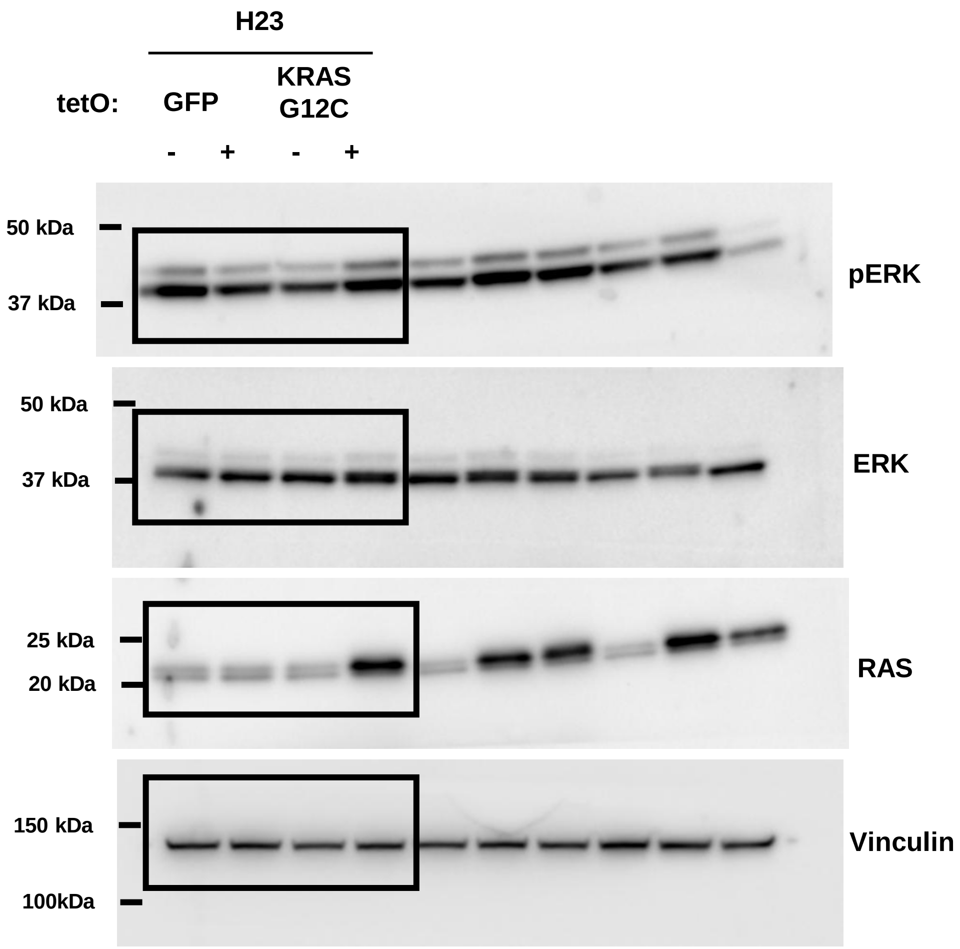

Supplementary Figure 15 Uncropped immunoblot images from indicated figure.

Uncropped Figure 5h

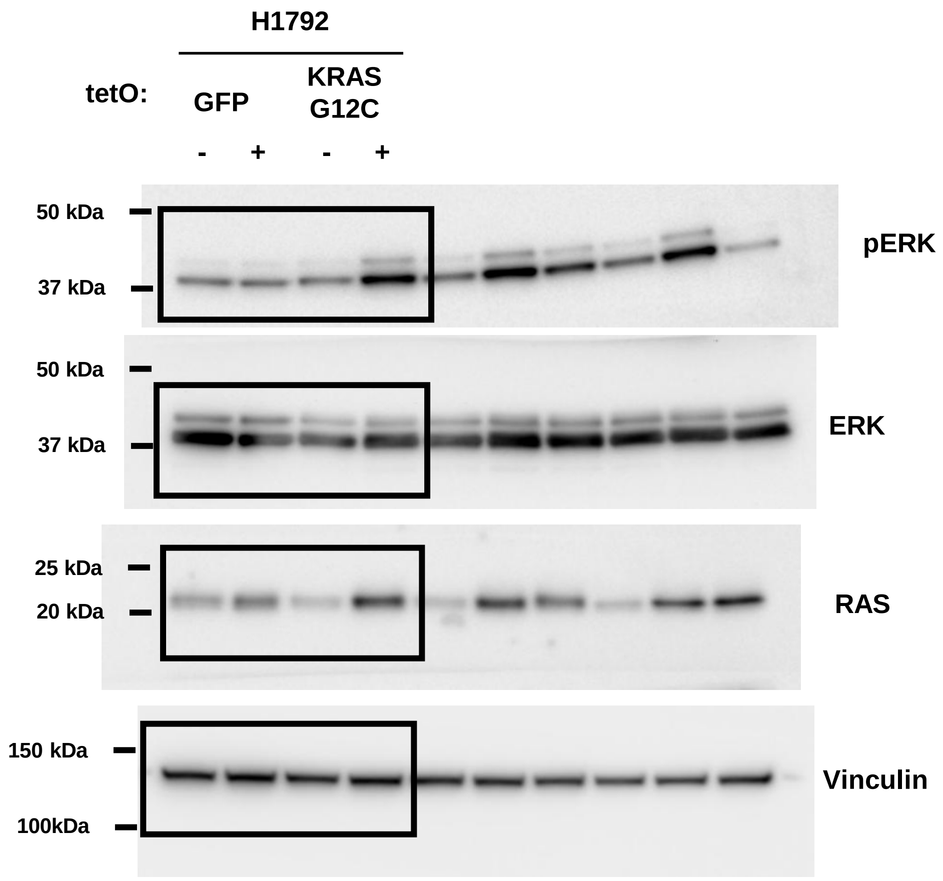

Supplementary Figure 16 Uncropped immunoblot images from indicated figure.

Uncropped Figure 5i

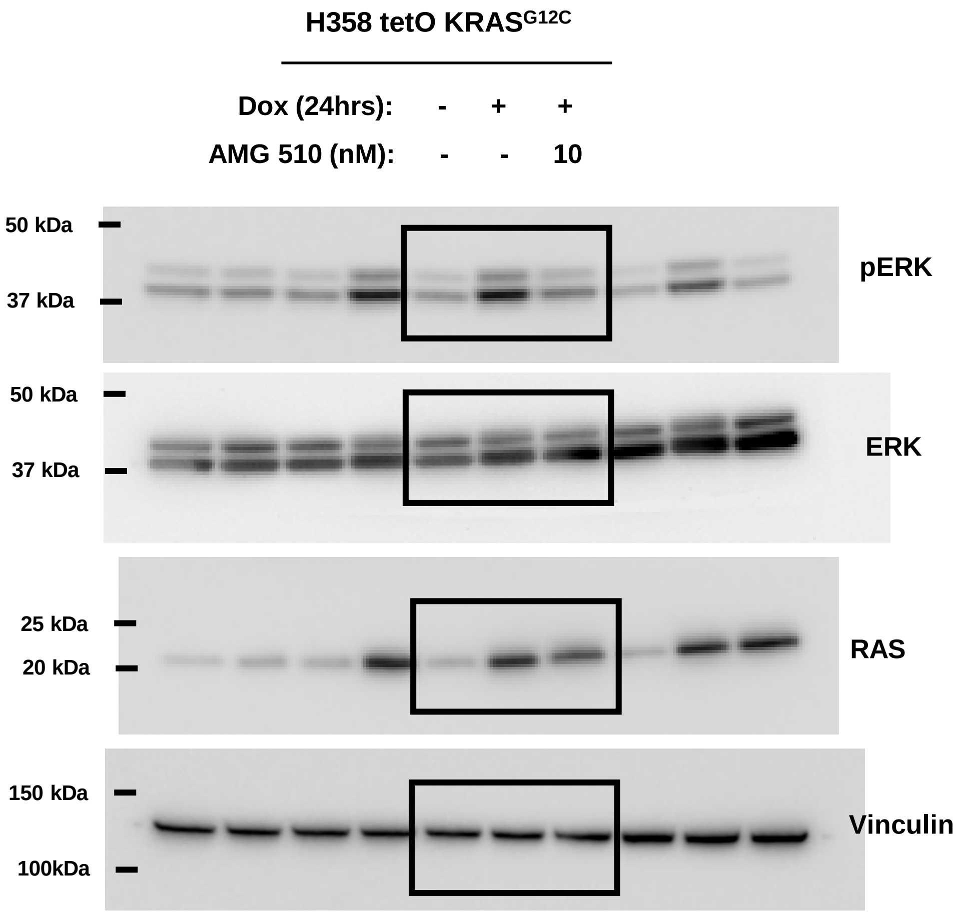

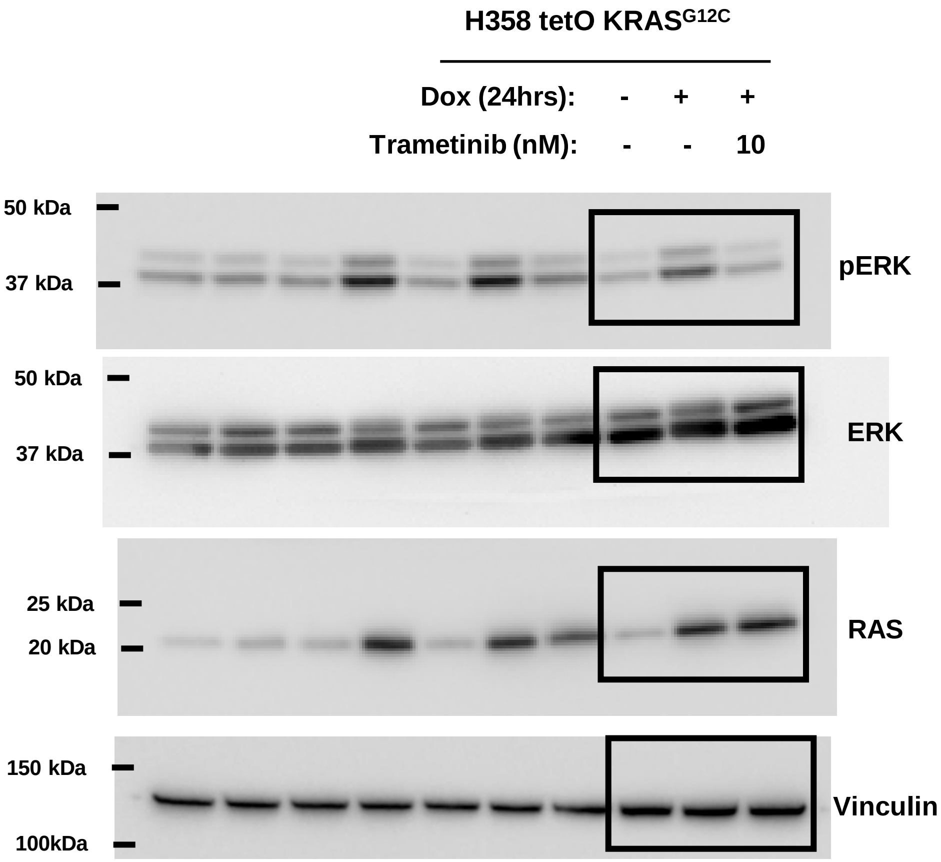

Supplementary Figure 17 Uncropped immunoblot images from indicated figure.

Uncropped Figure 5j

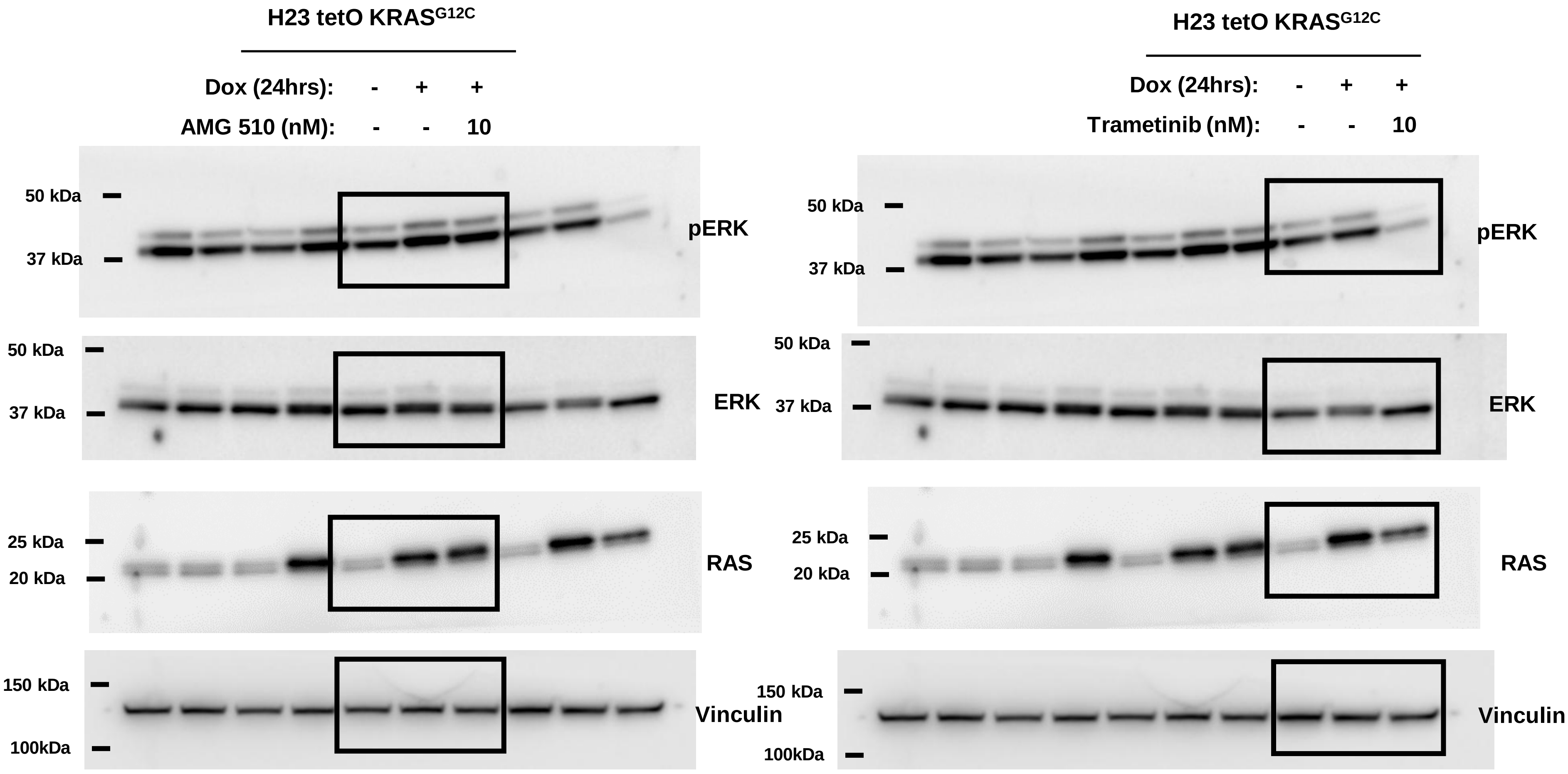

Supplementary Figure 18 Uncropped immunoblot images from indicated figure.

Uncropped Figure 5k

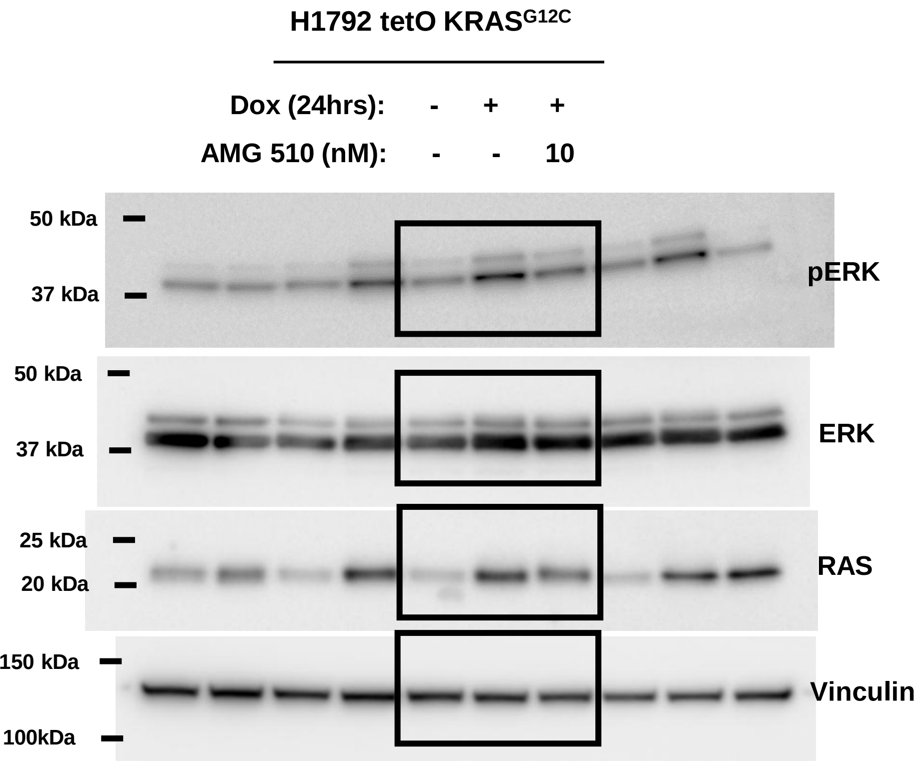

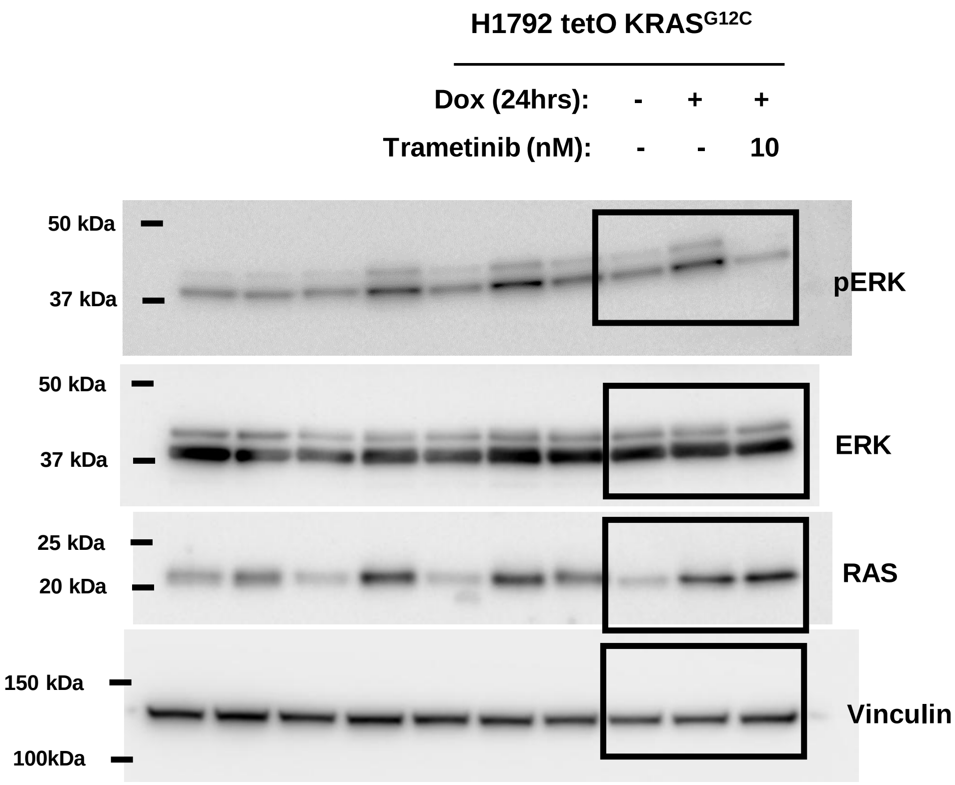

Supplementary Figure 19 Uncropped immunoblot images from indicated figure.

atgccgcccaaaacccccсgaaaaacggccgccacagcagcagctgcagcagcagaaccaccagcacctccacctccacctccacc
ggaagaagatccggaacaggacagcggcccggaggacctgcctctcgtcaggcttgagtttgaagaaacagaagaacctgattttactg
cattatgtcagaaattaaagataccagatcatgtcagagagagagcttggttaacttgggagaaagtttcatctgtggatggagtattgggag
gttatattcaaaagaaaaaggaactgtggggaatctgtatctttattgcagcagttgacctagatgagatgtcgttcacttttactgagctacag
aaaaacatagaaatcagtgtccataaattctttaacttactaaaagaaattgataccagtaccaaagttgataatgctatgtcaagactgttga
agaagtatgatgtattgtttgcactcttcagcaaattggaaaggacatgtgaacttatatatttgacacaacccagcagttcgatatctactgaa
ataaattctgcattggtgctaaaagtttcttggatcacatttttattagctaaaggggaagtattacaaatggaagatgatctggtgatttcatttc
agttaatgctatgtgtccttgactattttattaaactctcacctcccatgttgctcaaagaaccatataaaacagctgttatacccattaatggttc
acctcgaacacccaggcgaggtcagaacaggagtgcacggatagcaaaacaactagaaaatgatacaagaattattgaagttctctgta
aagaacacgagtgcaacatcgacgaggtgaagaacgtctacttcaagaacttcatcccttttatgaattctcttggacttgtaacatctaatgg
acttccagaggttgaaaatctttctaaacgatacgaagaaatttatcttaaaaataaagatctagatgcaagattatttttggatcatgataaaac
tcttcagactgattctatagacagttttgaaacacagagaacaccacgaaaaagtaaccttgatgaagaggtgaatgtaattcctccacaca
ctccagttaggactgttatgaacactatccaacaattaatgatgattttaaattcagcaagtgatcaaccttcagaaaatctgatttcctattttaa
caactgcacagtgaatccaaaagaaagtatactgaaaagagtgaaggatataggatacatctttaaagagaaatttgctaaagctgtggga
cagggttgtgtcgaaattggatcacagcgatacaaacttggagttcgcttgtattaccgagtaatggaatccatgcttaaatcagaagaaga
acgattatccattcaaaattttagcaaacttctgaatgacaacatttttcatatgtctttattggcgtgcgctcttgaggttgtaatggccacatata
gcagaagtacatctcagaatcttgattctggaacagatttgtctttcccatggattctgaatgtgcttaatttaaaagcctttgatttttacaaagtg
atcgaaagttttatcaaagcagaaggcaacttgacaagagaaatgataaaacatttagaacgatgtgaacatcgaatcatggaatcccttgc
atggctctcagattcacctttatttgatcttattaaacaatcaaaggaccgagaaggaccaactgatcaccttgaatctgcttgtcctcttaatct
tcctctccagaataatcacactgcagcagatatgtatctttctcctgtaagatctccaaagaaaaaaggttcaactacgcgtgtaaattctact
gcaaatgcagagacacaagcaacctcagccttccagacccagaagccattgaaatctacctctctttcactgttttataaaaaagtgtatcg
gctagcctatctccggctaaatacactttgtgaacgccttctgtctgagcacccagaattagaacatatcatctggacccttttccagcacac
cctgcagaatgagtatgaactcatgagagacaggcatttggaccaaattatgatgtgttccatgtatggcatatgcaaagtgaagaatatag
accttaaattcaaaatcattgtaacagcatacaaggatcttcctcatgctgttcaggagacattcaaacgtgttttgatcaaagaagaggagta
tgattctattatagtattctataactcggtcttcatgcagagactgaaaacaaatattttgcagtatgcttccaccaggcccсcctaccttgtcacc
aatacctcacattcctcgaagcccttacaagtttcctagttcaccccttacggattcctggagggaacatctatatttcacccсctgaagagtcca
tataaaatttcagaaggtctgccaacaccaacaaaaatgactccaagatcaagaatcttagtatcaattggtgaatcattcgggacttctgag
aagttccagaaaataaatcagatggtatgtaacagcgaccgtgtgctcaaaagaagtgctgaaggaagcaaccctcctaaaccactgaa
aaaactacgctttgatattgaaggatcagatgaagcagatggaagtaaacatctcccaggagagtccaaatttcagcagaaactggcaga
aatgacttctactcgaacacgaatgcaaaagcagaaaatgaatgatagcatggatacctcaaacaaggaagagaaatgc

Supplementary Figure 20 RB1 re-expression sequence.
