# Supplementary material for: MEK inhibitor resistance in lung adenocarcinoma is associated with addiction to sustained ERK suppression
Source: NPJ Precis Oncol. 2022 Nov 23;6:88. doi: 10.1038/s41698-022-00328-x (PMC9684561; doi:10.1038/s41698-022-00328-x)
Supplement: Supplementary file 2 — REPORTING SUMMARY [file 41698_2022_328_MOESM2_ESM.pdf]

nature portfolio

Corresponding author(s): William Lockwood
Last updated by author(s): Aug 23, 2022

Reporting Summary

Nature Portfolio wishes to improve the reproducibility of the work that we publish. This form provides structure for consistency and transparency in reporting. For further information on Nature Portfolio policies, see our Editorial Policies and the Editorial Policy Checklist.

Statistics

For all statistical analyses, confirm that the following items are present in the figure legend, table legend, main text, or Methods section.

n/a	Confirmed	
☐	☒	The exact sample size (*n*) for each experimental group/condition, given as a discrete number and unit of measurement
☐	☒	A statement on whether measurements were taken from distinct samples or whether the same sample was measured repeatedly
☐	☒	The statistical test(s) used AND whether they are one- or two-sided<br>*Only common tests should be described solely by name; describe more complex techniques in the Methods section.*
☒	☐	A description of all covariates tested
☒	☐	A description of any assumptions or corrections, such as tests of normality and adjustment for multiple comparisons
☐	☒	A full description of the statistical parameters including central tendency (e.g. means) or other basic estimates (e.g. regression coefficient) AND variation (e.g. standard deviation) or associated estimates of uncertainty (e.g. confidence intervals)
☐	☒	For null hypothesis testing, the test statistic (e.g. *F*, *t*, *r*) with confidence intervals, effect sizes, degrees of freedom and *P* value noted<br>*Give P values as exact values whenever suitable.*
☒	☐	For Bayesian analysis, information on the choice of priors and Markov chain Monte Carlo settings
☒	☐	For hierarchical and complex designs, identification of the appropriate level for tests and full reporting of outcomes
☐	☒	Estimates of effect sizes (e.g. Cohen's *d*, Pearson's *r*), indicating how they were calculated

Our web collection on statistics for biologists contains articles on many of the points above.

Software and code

Policy information about availability of computer code

Data collection	N/A
Data analysis	Statistical analyses were performed using GraphPad Prism version 8.2.1 (GraphPad Software, San Diego, CA, USA).

For manuscripts utilizing custom algorithms or software that are central to the research but not yet described in published literature, software must be made available to editors and reviewers. We strongly encourage code deposition in a community repository (e.g. GitHub). See the Nature Portfolio guidelines for submitting code & software for further information.

Data

Policy information about availability of data

All manuscripts must include a data availability statement. This statement should provide the following information, where applicable:

- Accession codes, unique identifiers, or web links for publicly available datasets
- A description of any restrictions on data availability
- For clinical datasets or third party data, please ensure that the statement adheres to our policy

The authors declare that the data supporting the findings of this study are available within the article and its supplementary information files.

Human research participants

Policy information about studies involving human research participants and Sex and Gender in Research.

Reporting on sex and gender	N/A
Population characteristics	N/A
Recruitment	N/A
Ethics oversight	N/A

Note that full information on the approval of the study protocol must also be provided in the manuscript.

Field-specific reporting

Please select the one below that is the best fit for your research. If you are not sure, read the appropriate sections before making your selection.

☒ Life sciences ☐ Behavioural & social sciences ☐ Ecological, evolutionary & environmental sciences

For a reference copy of the document with all sections, see nature.com/documents/nr-reporting-summary-flat.pdf

Life sciences study design

All studies must disclose on these points even when the disclosure is negative.

Sample size	Sample sizes for experiments with multiple replicates were calculated to achieve a power level of minimum 0.8 with a type 1 error rate of 0.05 based on effect sizes and standard deviations obtained in pilot studies.
Data exclusions	No data was excluded from analysis in this study.
Replication	In experiments where multiple independent replicates is indicated, cells of different passages were seeded on different days in the same experimental conditions. All attempts at replication were successful. Number of independent replicates for each experiment is indicated in the figure legends.<br>Effects of mutant KRAS on parental cell lines was validated across multiple biological replicates in H358, H23, H1792 and HCT116 cells.
Randomization	Cells were randomly sampled for seeding into treatment groups by pipetting from a homogeneous dilution of cells.
Blinding	Investigators were not blinded while performing experiments.

Reporting for specific materials, systems and methods

We require information from authors about some types of materials, experimental systems and methods used in many studies. Here, indicate whether each material, system or method listed is relevant to your study. If you are not sure if a list item applies to your research, read the appropriate section before selecting a response.

Materials & experimental systems

n/a	Involved in the study	
☐	☒	Antibodies
☐	☒	Eukaryotic cell lines
☒	☐	Palaeontology and archaeology
☒	☐	Animals and other organisms
☒	☐	Clinical data
☒	☐	Dual use research of concern

Methods

n/a	Involved in the study	
☒	☐	ChIP-seq
☒	☐	Flow cytometry
☒	☐	MRI-based neuroimaging

Antibodies

Antibodies used	The following antibodies were obtained from Cell Signaling Technologies (CST): pERK (9101S), ERK (4695S), pAKT (4060L), AKT (4691L), p-mTOR (5536S), mTOR (2983S), pFGFR Y653/654 (3471S), FGFR1 (9740S), pErbB3 Y1289 (4791S), ErbB3 (12708S), cleaved PARP (5625S), cleaved caspase 3 (9661S), cleaved caspase 7 (9491S), pH2AX (2577S), Rac1 (8631), cRAF (9422S), MEK1/2 (9122S), RAS (8955S), RB (9309S), E-cadherin (3195S), N-cadherin (13116S), vimentin (5741S), Snail (3879S), Slug (9585S), BiP (3183S), CHOP

(2895S), ATF4 (11815S), p-eIF2A (9721S), pJNK (4668S), Elk1 (9182S), c-Fos (9F6) (2250S), c-Myc (D84C12) (5605S), RSK1/RSK2/RSK3 (D7A2H) (14813S), Phospho-p90RSK S380 (D3H11) (11989S), c-Jun (60A8) (9165S), Phospho-c-Jun S73 (D47G9) (3270S), FRA1 (D80B4) (5281S), p27 Kip1 (D69C12) (3686T), p21 Waf1/Cip1 (12D1) (2947S), p16 INK4A (D7C1M) (80772S) & vinculin (E1E9V) (13901S). GAPDH (sc-47724) was obtained from Santa Cruz Biotechnology. TTF1 (MA5-16406) was obtained from ThermoScientific.

Validation

All antibodies purchased from CST were validated according to their in house validation protocols adapted from "A Proposal for Validation of Antibodies." Nature Methods (2016). Immunoblot specific validation techniques include:
-Examination of several cell lines and/or tissues of known expression levels allows accurate determination of species cross-reactivity and verifies specificity.
-Treatment of cell lines with growth factors, chemical activators or inhibitors, which induce or inhibit target expression, verifies specificity. Phosphatase treatment confirms phospho-specificity.
-The use of siRNA transfection or knockout cell lines verifies target specificity.
-Side-by-side comparison of lots to ensures lot-to-lot consistency.
-Optimal dilutions and buffers are predetermined, positive and negative cell extracts are specified, and detailed protocols are already optimized, saving valuable time and reagents.
Relevant citations for CST purchased antibodies are available on their website.

No validation protocols were provided by Santa Cruz for manufacture of GAPDH antibody (sc-47724). Citations for publications where GAPDH was previously used are available on their website.

The TTF1 (MA5-16406) antibody,purchased from Thermo Scientific, was validated according to their two part antibody validation standards consisting of target specific verification and functional application validation. Further details, along with citations for previous use of TTF1 are available online.

Eukaryotic cell lines

Policy information about cell lines and Sex and Gender in Research

Cell line source(s)

All cell lines were sourced from ATCC.

Authentication

H358 sg#4 parental and tramR were authenticated using STR profiling.

Mycoplasma contamination

All cells were routinely checked for mycoplasma using a PCR based approach as described in the methods section.

Commonly misidentified lines
(See ICLAC register)

No commonly misidentified cell lines were used in this study.
